# Diet and companionship modulate pain via a serotonergic mechanism

**DOI:** 10.1038/s41598-021-81654-1

**Published:** 2021-02-01

**Authors:** Huy Tran, Varun Sagi, Sarita Jarrett, Elise F. Palzer, Rajendra D. Badgaiyan, Kalpna Gupta

**Affiliations:** 1 Division of Hematology, Oncology and Transplantation, Department of Medicine, Vascular Biology Center, University of Minnesota, Minneapolis, MN USA; 2 Northwestern University, Evanston, IL USA; 3 Biostatistical Design and Analysis Center, Clinical and Translational Sciences Institute, University of Minnesota, Minneapolis, MN USA; 4 Department of Psychiatry, Long School of Medicine, University of Texas Health Science Center, San Antonio, Texas USA; 5 Hematology/Oncology, Department of Medicine, University of California, Irvine and Southern California Institute for Research and Education, VA Medical Center, 5901 East 7th St, Long Beach, CA 90822 USA

**Keywords:** Haematological diseases, Sickle cell disease

## Abstract

Treatment of severe chronic and acute pain in sickle cell disease (SCD) remains challenging due to the interdependence of pain and psychosocial modulation. We examined whether modulation of the descending pain pathway through an enriched diet and companionship could alleviate pain in transgenic sickle mice. Mechanical and thermal hyperalgesia were reduced significantly with enriched diet and/or companionship. Upon withdrawal of both conditions, analgesic effects observed prior to withdrawal were diminished. Serotonin (5-hydroxytryptamine, 5-HT) was found to be increased in the spinal cords of mice provided both treatments. Additionally, 5-HT production improved at the rostral ventromedial medulla and 5-HT accumulated at the dorsal horn of the spinal cord of sickle mice, suggesting the involvement of the descending pain pathway in the analgesic response. Modulation of 5-HT and its effect on hyperalgesia was also investigated through pharmaceutical approaches. Duloxetine, a serotonin-norepinephrine reuptake inhibitor, showed a similar anti-nociceptive effect as the combination of diet and companionship. Depletion of 5-HT through p-chlorophenylalanine attenuated the anti-hyperalgesic effect of enriched diet and companionship. More significantly, improved diet and companionship enhanced the efficacy of a sub-optimal dose of morphine for analgesia in sickle mice. These findings offer the potential to reduce opioid use without pharmacological interventions to develop effective pain management strategies.

## Introduction

Chronic pain occurs in a sizeable population of patients with a variety of chronic conditions^1–5^. Opioids remain the mainstay of chronic pain treatment^6^. Besides known side effects of opioids, including constipation and respiratory depression, social liabilities and premature deaths due to opioid abuse are a leading health concern^7–11^. A therapeutic strategy to effectively treat chronic pain without opioids remains a major unmet need^12,13^.

An effective alternative to opioids has not yet been found, partly because pain perception has both physiological and affective components^14^. A focus on medications for pain management has limited appreciation of the affective and psychological components of pain perception and their downstream biological pathways^15^. Affective state significantly alters pain perception and response to analgesics^16–18^. Thus, psychiatric conditions like depression and anxiety; and, environmental, cultural, emotional, and psychological factors influence pain perception by altering the affective state. Moreover, pain itself causes depression and anxiety^17,19–21^, which in turn lowers the pain threshold^22^. This interdependence of pain and affect makes treatment of chronic pain challenging^23^.

This challenge also provides an opportunity to control pain by modulating psychosocial environment. Psychological interventions are effective in controlling and even abolishing persistent pain^24^. Techniques such as hypnotism and mindfulness have been successfully used to modulate pain in sickle cell disease (SCD) and other conditions^25–27^. Success of these interventions led us to hypothesize that psychosocial enhancement could be an alternate strategy to treat chronic pain. This strategy, if successful, could eliminate widespread use of opioids that has become a major health concern all over the world.

SCD is a common genetic disorder, known for its complex and distinctive pain characteristics^9,28^. Acute pain in SCD is caused by aggregation of sickled red blood cells (RBC) blocking the vasculature, leading to vaso-occlusive crisis (VOC) and severe pain requiring hospitalization^9^. A significant proportion of people with SCD experience chronic pain, which may increase with age^28,29^. The global burden of SCD is primarily shouldered by communities with limited resources in Sub-Saharan Africa, Middle East, and India^30^. In the United States, the African-American population has the highest prevalence of SCD^31^. Due to low socioeconomic status of a substantial portion of the affected population and the life-long nature of unpredictable episodes of VOC and other comorbidities, chronic pain in SCD is accompanied by social stress^32^*.* Therefore, an unmet need is to develop a therapeutic strategy that controls pain effectively in SCD without using addictive drugs.

Serotonin (5-hydroxytryptamine, 5-HT) and dopamine (DA) are involved in affective and psychosocial regulation^33–36^ as well as nociceptive control^37^, suggesting that these neurotransmitters could provide a bridge between psychological and physiological pain control. Agents that elevate brain levels of 5-HT by blocking its re-uptake (selective 5-HT reuptake inhibitors) are the most commonly used antidepressant medications^38,39^. Agents, such as p-chlorophenylalanine (PCPA), which limit synthesis of 5-HT, have offered insights into the influence of 5-HT on affective regulation and pain^40–43^. DA elevates mood by modulating the reward system and by enhancing pleasure sensation^44^*.* Besides elevating mood, 5-HT and DA are also involved in modulating nociceptive stimuli via the descending pain pathway^37^. Enhanced levels of these neurotransmitters could therefore elevate mood and increase pain threshold, thus potentially reducing intensity and duration of pain^25–27^.

One approach to modulating psychosocial environment via increased levels of 5-HT and DA is through a diet enriched in amino acid precursors. Synthesis of 5-HT in the brain has been shown to be influenced by the availability of 5-HT precursors in the plasma which can be derived from dietary sources^45^. In particular, increasing plasma levels of tryptophan has been shown to positively correlate with levels of tryptophan crossing the blood brain barrier^45^, so that the rate of 5-HT synthesis inside the brain is elevated due to the increased saturation of tryptophan hydroxylase^45^. Consumption of tryptophan-rich hydrolyzed protein has been shown to increase brain 5-HT function and results in improved mood^46^. Providing enriched levels of dietary tyrosine has also been found to improve the synthesis rate of DA in the brain^47^, which can stimulate DA’s psychological effects^34^.

Significant involvement of the hypothalamic–pituitary–adrenal (HPA) axis in pain responses has also been established. One important class of molecules associated with this phenomenon is glucocorticoids, particularly corticosterone^48^. Increased corticosterone in plasma as a result of HPA axis activation can induce pain-like behaviors in rodents^49,50^. Moreover, elevated expression of glucocorticoid receptor is observed in pain-transmitting neurons after nerve injury in rodents^51,52^. These findings suggest that corticosterone plays an important role in pain regulation in many conditions, potentially impacting the efficacy of analgesic medications.

This study examined whether an enriched diet and/or companionship can suppress pain in humanized transgenic sickle mice. Since humanized transgenic sickle mice and SCD patients have similar clinical features and pain characteristics, the results could have significant translational value^53^. The study also examined whether the modulation of pain by enriched diet and/or companionship is mediated by the 5-HT system.

## Results

**Figure 1:**
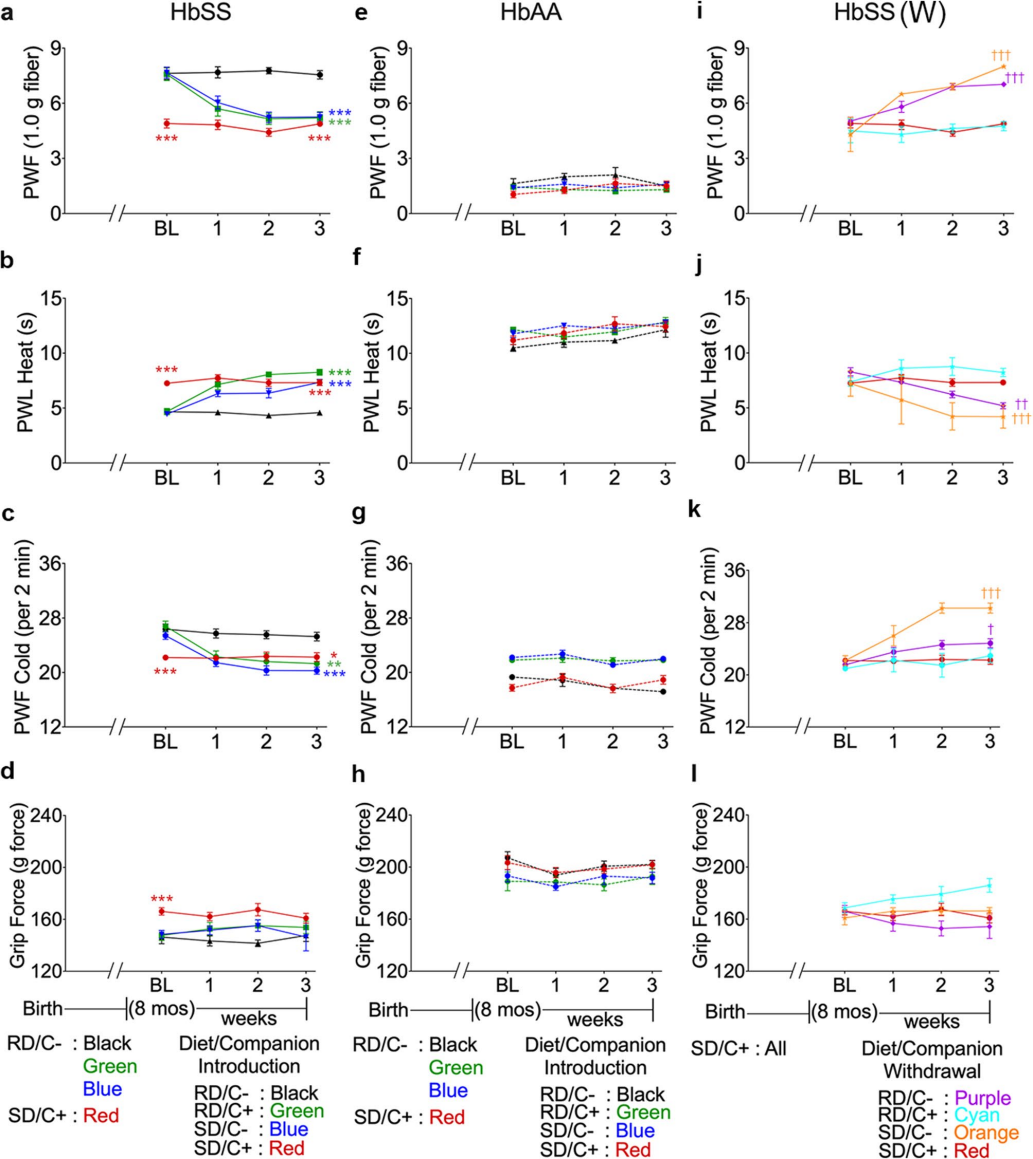
Effect of enriched diet and companionship on hyperalgesia in male sickle and control mice. Mice were fed specific diets from birth until approximately 8 months of age as indicated on the left side of each figure. Diet and/or companion introduction or withdrawal was done for three weeks following baseline testing; specific treatment groups are listed on the right side of the figure. Hyperalgesia measures for all mice are shown for, mechanical hyperalgesia as PWF in response to 1.0 g von Frey filaments; heat hyperalgesia as PWL in response to a heat stimulus; cold hyperalgesia as PWF per 2 min on a cold plate at 4 °C and deep tissue/musculoskeletal hyperalgesia as grip force exerted by forelimbs. (**a**–**d**) Male sickle mice (HbSS) given sickle or regular diet in the presence or absence of a female companion. (**e**–**h**) Male control mice (HbAA) given sickle or regular diet in the presence or absence of a female companion. (**i**–**l**) Male sickle mice (HbSS W) fed sickle diet in the presence of a female companion followed by withdrawal of sickle diet replaced with regular diet and/or withdrawal of the female companion. Regular diet without companionship, RD/C−, nSS = 12, nAA = 8; regular diet with companionship, RD/C+, nSS = 10, nAA = 10; sickle diet without companionship, SD/C−, nSS = 13, nAA = 10; sickle diet with companionship, SD/C+, nSS = 20, nAA = 11; withdrawn from sickle diet, W RD/C+, nSS = 5; withdrawn from companionship, W SD/C−, nSS = 6; withdrawn from sickle diet and companionship, W RD/C−, nSS = 20, nAA = 8; mixed linear models with Tukey’s post-hoc test, *p < 0.05, **p < 0.01, ***p < 0.001 compared to HbSS RD/C−; ^†^*p* < 0.05, ^††^*p* < 0.01, ^†††^*p* < 0.001 compared to HbSS SD/C+. PWF, paw withdrawal frequency; PWL, paw withdrawal latency.

**Study groups and their designation**. We divided male mice randomly into 2 major treatment groups. In the first group, we used 4 different conditions based on feeding regular rodent diet (RD) or special sickle diet (SD) and the absence or presence of a female companion (C) as shown in Fig. 1. (i) Since weaning, males on RD were housed singly without a companion, called RD/C−, (ii) Since weaning males on RD were housed singly and a female companion was introduced at 8 months of age for 3 weeks to examine the effect of companionship, called RD/C+, (iii) Since weaning males on RD were housed singly and SD was introduced at 8 months of age for 3 weeks to examine the effect of diet, called SD/C−, and (iv) Since weaning males on SD were housed with a female companion, called SD/C+. The second group was created to examine the impact of withdrawal (W) of sickle diet and/or companionship, using male mice that were fed the sickle diet and housed with a companion (SD/C +) since weaning up to the beginning of the second study period as follows: (v) withdrawal of SD and continuation of RD and withdrawal of companion, called W RD/C−, (vi) withdrawal of SD and continuation of RD in the presence of companion, called W RD/C+, and (vii) continuation of SD and withdrawal of companion, called W SD/C−. These conditions were created to simulate stressful conditions of undernutrition and loneliness in group 1 faced throughout life by many individuals, whereas the 2nd group was intended to simulate sudden adverse conditions that may occur spontaneously, such as death of a loved one leading to loneliness and/or sudden loss of resources leading to poor nutrition.

### Companionship and improved nutrition reduce hyperalgesia

#### Effect of diet and/or companionship on hyperalgesia in male sickle mice

We observed significantly reduced mechanical, heat, and cold hyperalgesia after three weeks of treatment in male sickle mice in the RD/C+, SD/C−, and SD/C+ treatment groups compared to the RD/C− group (Fig. 1a–c). However, we did not observe a significant reduction in musculoskeletal hyperalgesia after three weeks of treatment for mice in the RD/C+, SD/C−, and SD/C+ treatment groups compared to the RD/C− group (Fig. 1d). Mice in the RD/C+ and SD/C− groups showed levels of hyperalgesia comparable to those in the SD/C+ group after just three weeks of treatment. This is significant as the SD/C+ treatment group was maintained on the sickle diet and companionship since weaning, while those in the RD/C+ and SD/C− groups were only provided enriched diet or a companion for three weeks after being on the regular diet without companionship before treatment initiation. Control mice expressing normal human hemoglobin A, which do not have constitutive hyperalgesia, did not show a significant change in hyperalgesia with different treatments (Fig. 1e–h).These data show that improving the nutritional requirements and companionship can independently reduce pain/hyperalgesia in sickle mice.

#### Effect of withdrawal of nutritious diet and/or female companion on hyperalgesia in male sickle mice

Withdrawal of companionship alone (W SD/C −) or both companionship and the sickle diet (W RD/C −) for three weeks led to a significant increase in mechanical, heat, and cold hyperalgesia (Fig. 1i–k). Withdrawal of only the sickle diet (W RD/C +) for three weeks, however, did not lead to a significant increase in mechanical, heat or cold hyperalgesia. Withdrawal of companionship had a more significant impact on mechanical, heat, and cold hyperalgesia than did the sickle diet. For musculoskeletal hyperalgesia, withdrawal of companionship (W SD/C −), the sickle diet (W RD/C +), or both (W RD/C −) for three weeks did not lead to a significant increase in hyperalgesia (Fig. 1l). Together, these data suggest that sudden loneliness in male sickle mice contributes to hyperalgesia irrespective of nutrition.

#### Effect of diet and/or companionship on hyperalgesia in female sickle mice

Several challenges including irregular menstrual cycles (which influence pain), and pregnancy risk upon addition of a male companion interfered with examining hyperalgesia in female sickle mice. To prevent the risk of alterations due to pregnancy, older female mice at a mean age of approximately 9 months were tested. However, some mice still became pregnant and significant intra-group variability in hyperalgesia precluded from drawing conclusions (Fig. S1).

### Nutrition and companionship activated the descending pain pathway

#### Effect of diet and/or companionship on 5-HT and DA in whole spinal cord lysates of male sickle mice

**Figure 2:**
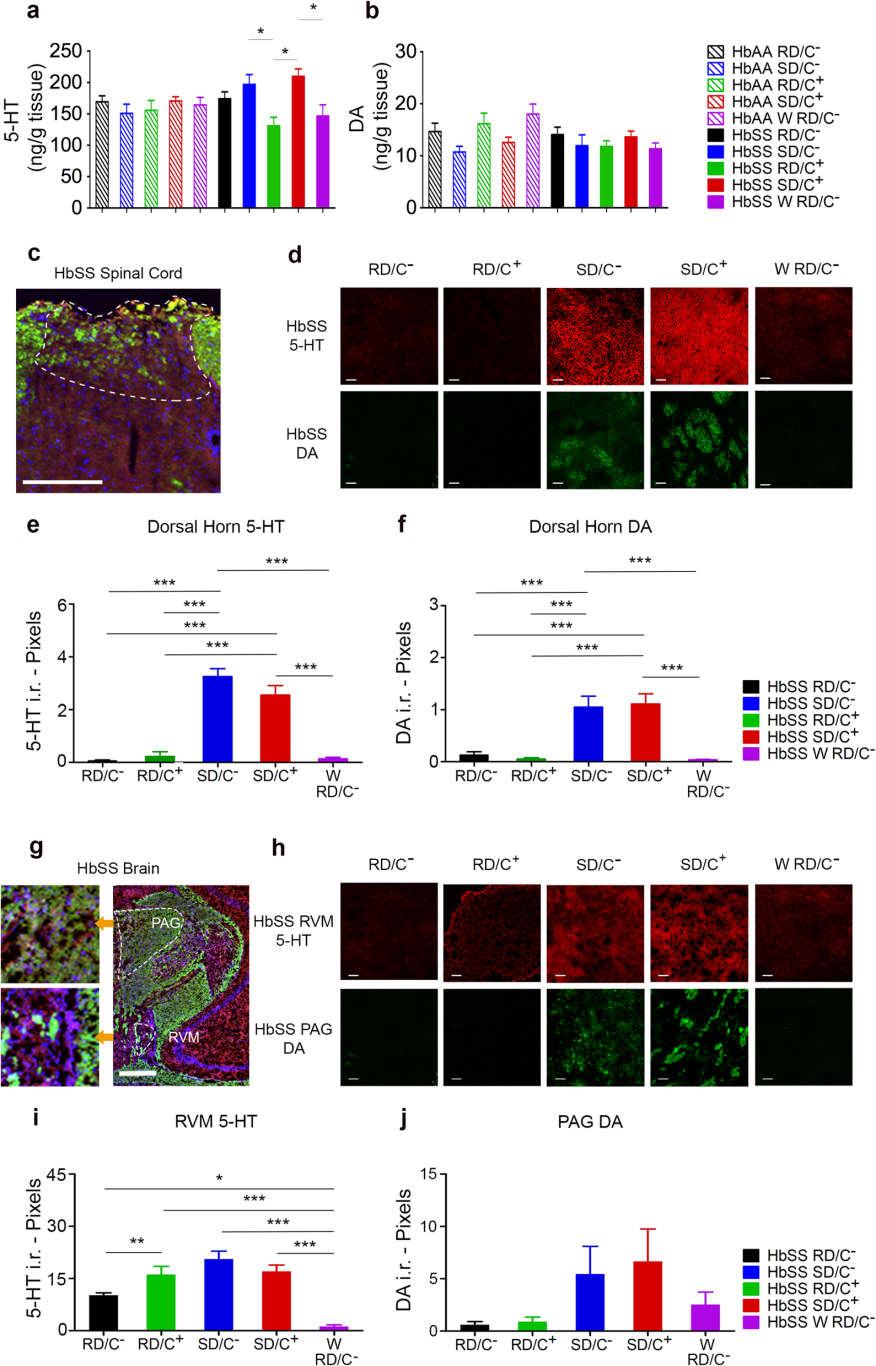
Effect of enriched diet and companionship on levels of 5-HT and DA in the central nervous system of male sickle mice. (**a**) Level of 5-HT in whole spinal cord. (**b**) Level of DA in whole spinal cord. (**c**, **d**) Confocal images showing distribution of 5-HT and DA in the dorsal horn. (**e**) Quantification of 5-HT level in the dorsal horn. (f) Quantification of DA level in the dorsal horn. (**g**, **h**) Confocal images showing distribution of 5-HT and DA in the brain. (**i**) Quantification of 5-HT level in the RVM area of the brain. (**j**) Quantification of DA in the PAG area of the brain. Male HbSS-BERK and HbAA-BERK mice between 8 and 10 months of age were used. Regular diet without companionship, RD/C−, **a**, **b**: nSS = 9, nAA = 8, **c**–**f**: nSS = 7, **g**–**j**: nSS = 6; regular diet with companionship, RD/C+, **a**, **b**: nSS = 6, nAA = 10, **c–f**: nSS = 8, **g–j**: nSS = 6; sickle diet without companionship, SD/C−, **a**, **b**: nSS = 9, nAA = 9, **c–f**: nSS = 9; **g**–**j**: nSS = 6; sickle diet with companionship, SD/C+, **a**, **b**: nSS = 7, nAA = 9, **c**–**f**: nSS = 9, **g–j**: nSS = 8; withdrawn from sickle diet and companionship, W RD/C−, **a**, **b**: nSS = 10, nAA = 7, **c**–**f**: nSS = 7, **g**–**j**: nSS = 6; one-way ANOVA with Bonferroni’s post-hoc test, *p < 0.05, **p < 0.01, ***p < 0.001. Scale bar (**c** and **g**) 500 µm, (**d** and **h**) 20 µm. RVM, rostral ventromedial; PAG, periaqueductal grey; i.r, immunoreactivity.

High Performance Liquid Chromatography (HPLC) of neurotransmitters in spinal cord lysate confirmed an increase in 5-HT in whole spinal cords of sickle mice in the SD/C− and the SD/C+ treatment groups compared to those in the RD/C− group, though these increases were not significant (Fig. 2a). We observed a reduction of 5-HT in sickle mice in the RD/C+ group compared to those in the RD/C− treatment groups, but this decrease was also not significant (Fig. 2a). However, we found significantly higher levels of 5-HT in whole spinal cords of mice in the SD/C− and SD/C+ groups compared to the RD/C+ treatment group, suggestive of a contribution of sickle diet to elevated 5-HT independent of companionship. This contribution of sickle diet is further validated by a significantly lower 5-HT level in the W RD/C− group when compared to the SD/C+ group (Fig. 2a). DA levels in whole spinal cords of sickle mice did not differ significantly between treatment groups (Fig. 2b). Thus, sickle diet contributes to 5-HT in the spinal cord of male sickle mice. It is likely that a greater effect occurs in the dorsal horn where neurotransmitters are released and therefore it may not be truly reflected in whole spinal cord lysates.

#### Effect of diet and/or companionship on 5-HT and DA in the dorsal horn of the spinal cord in male sickle mice

Since the descending pain pathway releases neurotransmitters in the dorsal horn, we also analyzed 5-HT and DA levels in this structure^37^. Significantly lower 5-HT immunoreactivity was observed in the dorsal horn of spinal cords in sickle mice in the RD/C−, RD/C+ and W RD/C− groups compared to either the SD/C− or SD/C+ groups (Fig. 2e). Similarly, we observed significantly lower DA immunoreactivity in the dorsal horn of spinal cords in sickle mice in the RD/C−, RD/C+ and W RD/C− groups compared to either the SD/C− or SD/C+ groups (Fig. 2f). These data suggest that SD contributes to 5-HT and DA release in the descending pain inhibitory pathways.

#### Effect of diet and/or companionship on 5-HT and DA in the higher brain regions of male sickle mice

Next, we examined 5-HT and DA immunoreactivity in the higher brain regions involved in the descending anti-nociceptive pathway. Release of 5-HT in the rostral ventromedial medulla (RVM) was significantly increased in sickle mice in the RD/C+ group compared to the RD/C− group (Fig. 2i). An increase in 5-HT was also observed in the RVM of sickle mice in the SD/C− and SD/C+ groups compared to the RD/C− group, but this increase was not statistically significant (Fig. 2i). The W RD/C− group had significantly lower 5-HT in the RVM compared to the RD/C−, RD/C+, SD/C−, and SD/C+ groups (Fig. 2i). The DA levels in the periaqueductal gray (PAG) for sickle mice did not differ significantly between any pair of treatment groups (Fig. 2j). These data demonstrate that companionship positively impacted the RVM by significantly increasing 5-HT, and withdrawal of both sickle diet and companionship has a significant effect on reducing 5-HT in the RVM.

### Effect on circulating corticosterone levels in response to diet and companionship

Stress is an aggravating factor in several chronic conditions and has been associated with fluctuations in pain level in patients dealing with chronic pain^54^. We therefore examined whether improved diet and companionship could lead to changes in levels of corticosterone, a stress indicator, in sickle mice.

**Figure 3:**
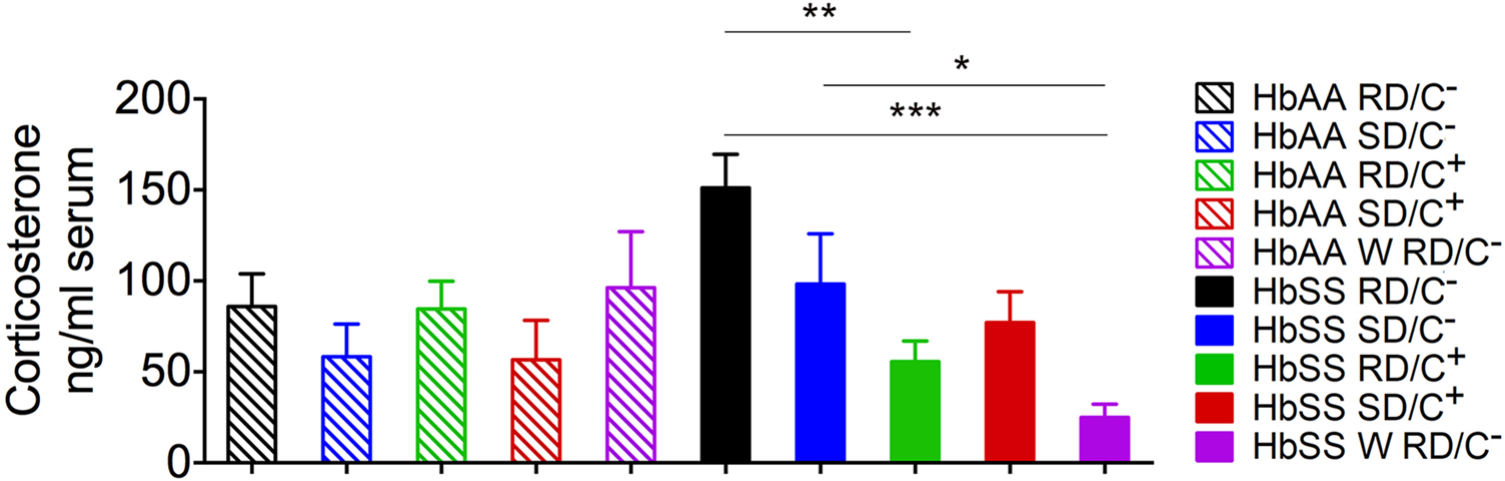
Effect of enriched diet and companionship on levels of corticosterone in serum of male sickle mice*.* As described in Fig. 1(a–h) male mice were treated with regular or sickle diet in the absence or presence of a female companion for 3 weeks, and a group of male mice fed sickle diet in the presence of a companion upto 8 months of age were withdrawn (W) from sickle diet and the companion for 3 weeks. Blood was drawn after 3 weeks of treatments and after 3 weeks of withdrawal for corticosterone analysis. Regular diet without companionship, RD/C−, nSS = 9, nAA = 9; regular diet with companionship, RD/C+, nSS = 9, nAA = 9; sickle diet without companionship, SD/C−, nSS = 10, nAA = 9; sickle diet with companionship, SD/C+, nSS = 8, nAA = 9; withdrawn from sickle diet and companionship, W RD/C−, nSS = 9, nAA = 10; one-way ANOVA with Bonferroni’s post-hoc test, *p < 0.05, **p < 0.01, ***p < 0.001.

Corticosterone levels were significantly reduced in sickle mice in the RD/C+ group compared to those in the RD/C− group (Fig. 3). Reduced levels of corticosterone were also observed in the SD/C− and SD/C+ groups compared to RD/C−, but these were not statistically significant (Fig. 3). Lower corticosterone levels were found in sickle mice in the W RD/C− group compared to the SD/C+ group, although this decrease was not statistically significant. The sickle mice in the W RD/C− group, however, did show a significantly lower level of corticosterone compared to the SD/C− or RD/C− treatment groups (Fig. 3). Such a significant drop in corticosterone level suggests impaired compensatory stress response as a result of withdrawal of SD and companionship^55,56^.

### Serotonin-norepinephrine reuptake inhibitor (SNRI, 5-HT-NE), duloxetine, ameliorates thermal hyperalgesia in sickle mice

Since increased 5-HT levels, resulting from improved nutrition and companionship, were associated with analgesia in sickle mice, we examined whether a pharmacological strategy to increase 5-HT levels in the nervous system could also ameliorate hyperalgesia in sickle mice. We used duloxetine, an SNRI, which has been shown to be effective in reducing pain and improving health-related quality of life (HRQoL) in patients with knee pain due to arthritis in a Phase III clinical trial^57^.

**Figure 4:**
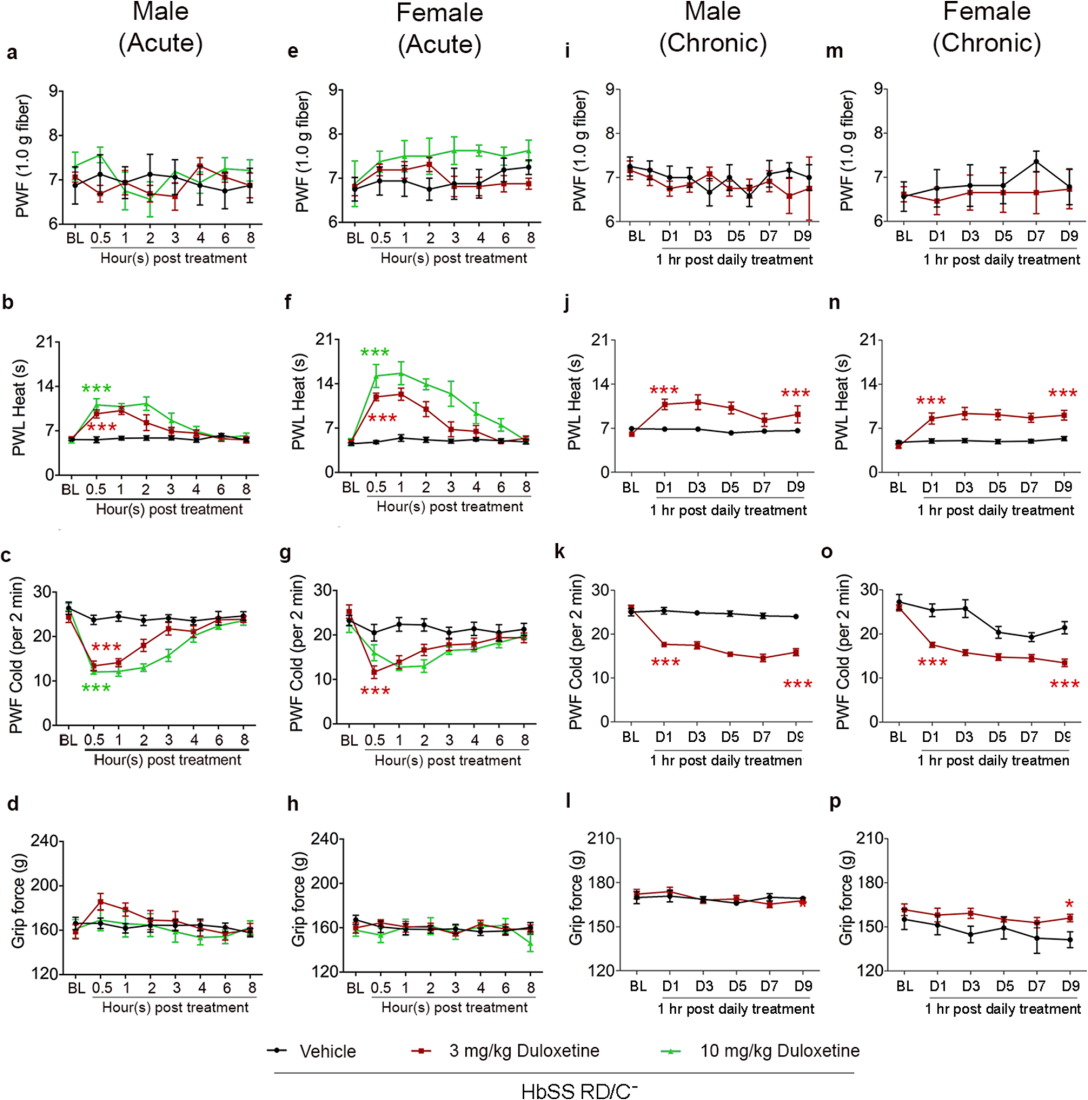
Effect of acute and chronic duloxetine treatment on hyperalgesia in male and female sickle mice. All mice were fed regular diet and housed singly without a companion. Hyperalgesia measures for all mice are shown for, mechanical hyperalgesia as PWF in response to 1.0 g von Frey filaments; heat hyperalgesia as PWL in response to a heat stimulus; cold hyperalgesia as PWF per 2 min on a cold plate at 4 °C and deep tissue/musculoskeletal hyperalgesia as grip force exerted by forelimbs. Effect of an *acute* single dose of duloxetine at 3 and 10 mg/Kg or vehicle (**a**–**h**) and of chronic administration of 3 mg/Kg/day duloxetine or vehicle for 9 days (**i**–**p**), in male and female sickle mice on hyperalgesia. Acute duloxetine treatment in male sickle mice (**a**–**d**), nSS vehicle = 8, nSS 3 mg/kg duloxetine = 8, nSS 10 mg/kg duloxetine = 8; Acute duloxetine treatment in female sickle mice (**e**–**h**), nSS vehicle = 8, nSS 3 mg/kg duloxetine = 8, nSS 10 mg/kg duloxetine = 4; Chronic duloxetine treatment in male sickle mice (**i**–**l**), nSS vehicle = 6, nSS 3 mg/kg duloxetine = 6; Chronic duloxetine treatment in female sickle mice (**m**–**p**), nSS vehicle = 8, nSS 3 mg/kg duloxetine = 13; mixed linear models with Tukey’s post-hoc test, *p < 0.05, ***p < 0.001 compared to vehicle. D1, D3, D5, D7, and D9, day 1, 3, 5, 7, and 9 respectively; PWF, paw withdrawal frequency; PWL, paw withdrawal latency.

Male and female sickle mice fed RD and housed without a companion (RD/C −) uniformly showed a significant decrease in heat and cold hyperalgesia 30 min after treatment with 3 and 10 mg/kg duloxetine, returning to baseline levels 6–8 h post injection (Fig. 4b–c, f–g). No significant change in mechanical or musculoskeletal hyperalgesia was observed in either male or female sickle with one-time duloxetine treatment. After 9-day treatment with duloxetine at 3 mg/kg, the anti-hyperalgesic response for heat and cold hyperalgesia remained without causing tolerance in both male and female sickle mice (Fig. 4j–k, n–o). Additionally, long term treatment with 3 mg/kg duloxetine led to significantly lower musculoskeletal hyperalgesia in female sickle mice, which was not observed with one-time treatment (Fig. 4p). These data suggest intervention with 5-HT increasing pharmacologics can ameliorate hyperalgesia in both male and female sickle mice with a greater effect on female mice without causing tolerance.

### p-chlorophenylalanine (PCPA), inhibitor of tryptophan hydroxylase, a rate limiting enzyme in the biosynthesis of 5-HT, induces hyperalgesia in sickle mice

A decreased level of 5-HT and an associated increase in hyperalgesia were observed in sickle mice following withdrawal of both the sickle diet and companionship (W RD/C −). We tested whether pharmacologically targeted endogenous depletion of 5-HT could also increase hyperalgesia.

**Figure 5:**
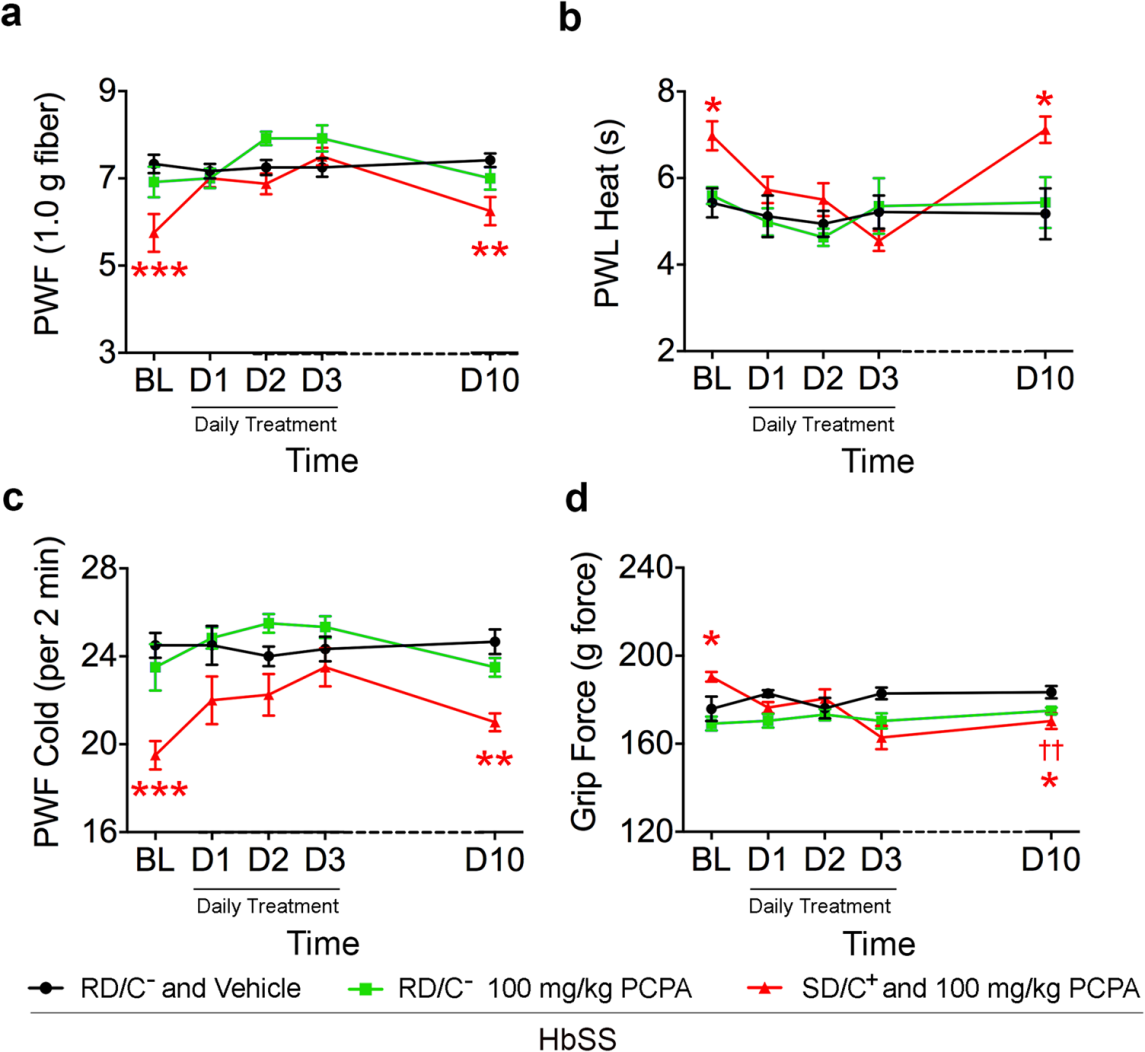
Effect of p-chlorophenylalanine (PCPA) on hyperalgesia in male sickle mice. Male sickle mice fed regular diet and housed singly without a companion (RD/C −) were treated with vehicle or 100 mg/Kg PCPA/day and mice fed sickle diet and housed with a female companion were treated with 100 mg/Kg PCPA/day. Hyperalgesia measures for all mice are shown for, mechanical hyperalgesia as PWF in response to 1.0 g von Frey filaments; heat hyperalgesia as PWL in response to a heat stimulus; cold hyperalgesia as PWF per 2 min on a cold plate at 4 °C and deep tissue/musculoskeletal hyperalgesia as grip force exerted by forelimbs. Male HbSS-BERK mice between 8 and 10 months of age were used. Regular diet without companionship, RD/C−, nSS vehicle = 8, nSS 100 mg/kg PCPA = 13; sickle diet with companionship, SD/C+, nSS 100 mg/kg PCPA = 4; mixed linear models with Tukey’s post-hoc test, *p < 0.05, **p < 0.01, ***p < 0.001 compared to RD/C− with vehicle; ^††^*p* < 0.01 compared to SD/C+ at baseline. D1, D2, D3, D10, day 1, 2, 3, and 10 respectively; PWF, paw withdrawal frequency; PWL, paw withdrawal latency.

At baseline, mechanical, heat, cold and musculoskeletal hyperalgesia in sickle mice in the SD/C+ group were significantly lower compared to sickle mice in the RD/C− group (Fig. 5). After three days of PCPA treatment, mechanical, heat, cold, and musculoskeletal hyperalgesia in SD/C+ sickle mice were elevated to the level of RD/C− sickle mice. One week after discontinuing PCPA treatment, mechanical, heat, and cold hyperalgesia in SD/C+ sickle mice returned to their baseline levels, but musculoskeletal hyperalgesia remained increased when compared to SD/C+ baseline before PCPA treatment (Fig. 5). These data validate the role of 5-HT in ameliorating hyperalgesia and the effectiveness of SD and companionship in elevating 5-HT.

### Nutritional diet and companionship improves opioid analgesia

High doses of opioids are often required to treat sickle pain compared to analogous pain in other conditions^28^. Therefore, we examined whether modulation of the descending pain pathway via improved diet and companionship could reduce opioids needed to treat pain in sickle mice.

**Figure 6:**
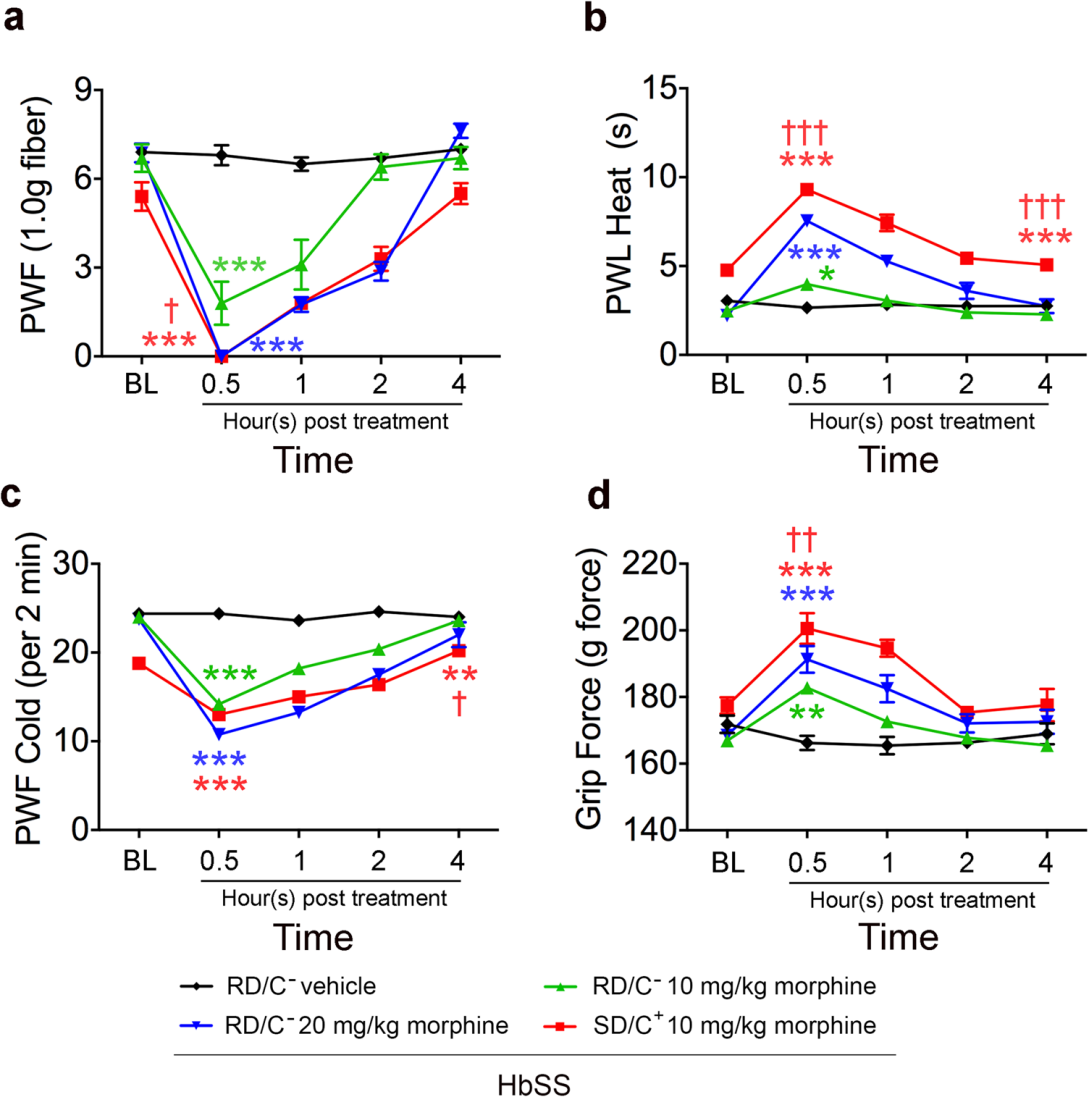
Effect of enriched diet and companionship on analgesic effect of sub-optimal dose of morphine in male sickle mice. Male sickle mice housed singly without a companion fed with regular diet (RD/C −) were treated with vehicle, and 3 or 10 mg/Kg morphine sulfate. Another group of male mice fed sickle diet and housed with a female companion were treated with 10 mg/kg morphine sulfate. Hyperalgesia measures for all mice are shown for, mechanical hyperalgesia as PWF in response to 1.0 g von Frey filaments; heat hyperalgesia as PWL in response to a heat stimulus; cold hyperalgesia as PWF per 2 min on a cold plate at 4 °C and deep tissue/musculoskeletal hyperalgesia as grip force exerted by forelimbs. Male HbSS-BERK mice between 8 and 10 months of age were used. Regular diet without companionship, RD/C−, nSS vehicle = 5, nSS 10 mg/kg morphine = 5, nSS 20 mg/kg morphine = 5; sickle diet with companionship, SD/C+, nSS 10 mg/kg morphine = 5; mixed linear models with Tukey’s post-hoc test, *p < 0.05, **p < 0.01, ***p < 0.001 compared to RD/C− with vehicle; ^†^*p* < 0.05, ^††^*p* < 0.01, ^†††^*p* < 0.001 compared to RD/C− with 10 mg/kg morphine. *PWF* paw withdrawal frequency; *PWL* paw withdrawal latency.

A sub-optimal dose of 10 mg/kg of morphine in RD/C− sickle mice was less effective in reducing mechanical, heat, cold, and deep tissue hyperalgesia compared to the higher dose of 20 mg/kg (Fig. 6). However, hyperalgesia was significantly ameliorated by a sub-optimal dose (10 mg/kg) of morphine in SD/C+ sickle mice (Fig. 6). RD/C− sickle mice required 20 mg/kg of morphine to achieve a comparable effect, twice the dose required by SD/C+ sickle mice (Fig. 6). Therefore, improved nutrition and companionship which reduce stress improve the response to opioid therapy.

## Discussion

Psychological factors are known to alter pain perception in a variety of conditions^5,19,58–63^. We observed that both the SD and companionship individually and in combination reduced hyperalgesia in sickle mice. This suggests that persistent chronic pain can be attenuated by enriching environmental conditions. We also found that removing companionship alone or both companionship and the SD led to restored hyperalgesia. This observation is consistent with reports of elevated pain in response to negative emotion^64–66^ and poor diet^11^ and reduction in opioid overdose with behavioral interventions^67^. It is believed that poor nutrition alters the leptin/ghrelin hormone balance^68^, resulting in development of psychiatric symptoms such as anxiety and depression^69,70^. Therefore, HRQoL including malnutrition, loneliness, stigmatization, etc. and adverse life events can sustain or worsen hyperalgesia.

Multiple neurotransmitters including 5-HT and DA are involved in processing pain sensation and perception. These neurotransmitters modulate pain perception^71–75^. In this context, it is significant that we observed increased 5-HT concentration in the spinal cord of the group that received enriched diet and companionship, particularly because the higher concentration was observed in the dorsal horns, where signals transmitted from the descending pain pathway are modulated. Since higher 5-HT concentration is associated with elevated mood^76–78^, it appears that the positive mood induced by the SD and companionship increased 5-HT production which in turn induced analgesia.

The decreased level of 5-HT in sickle mice lacking SD in the RD/C− and RD/C+ treatment groups as compared to the RD/C+ and SD/C+ groups respectively demonstrates the important role of diet in production of 5-HT. Moreover, when paired with companionship (SD/C +), enriched diet elevated 5-HT to the highest level among treatment groups. Therefore, nutrition can modulate 5-HT in response to pain and/or distress.

The observation of increased 5-HT in the SD/C+ group is an important finding. It suggests that pain modulation induced by the descending pathways is mediated by the 5-HT releasing system. To our knowledge, this is the first study to demonstrate 5-HT activating supraspinal control of nociception in the context of SCD.

Depletion of 5-HT in the brain by PCPA was previously shown to decrease pain threshold and unresponsiveness to morphine in healthy rodents^42,43^. Similar hyperalgesia-induced effects were observed in sickle mice who were previously in the SD/C+ treatment group after short-term treatment with PCPA, implying the relationship between reduced 5-HT and chronic pain in sickle mice. These data support the role of 5-HT in modulation of sickle pain.

We confirmed the role of 5-HT in mediating analgesia by enhancing 5-HT availability in the synapses. Using a SNRI, duloxetine, produced an analogous effect to that of improved diet and companionship in sickle mice, which suggests that these benefits could be induced with SNRI drug treatment. Since pain in females is more challenging to manage than in males^79–82^, therapies targeting the descending pain pathway may improve analgesic outcome in females. In SCD, females and older subjects were found to require neuropathic pain treatment and had longer hospital stays compared to males and younger patients^83^. Therefore, targeting of the descending serotonergic pathway may be beneficial in treating pain in these vulnerable populations of SCD.

We also observed reduced corticosterone levels in sickle mice with sickle diet and/or companionship. Since corticosterone is involved in stress response, this observation is consistent with reported stress-relieving effects of satisfying food^84,85^ and social bonding^86,87^. Stress has been known for decades to enhance pain perception, especially in patients having chronic pain^88–91^. Our observation of consistently high hyperalgesia in sickle mice lacking enriched diet and companionship further supports this concept. It is likely that the stress-relieving effect of enriched diet and companionship contributes to analgesic responses in sickle mice. A study of 121 married/partnered patients with colorectal cancer demonstrated that intimacy moderated the association between processing and depressive symptoms^92^. It was found that relatively high intimacy relationships were associated with lower depression and that the quality of relationship and emotional approach may enhance coping efforts. Therefore, strategies to reduce stress and improve emotion in patients may be helpful in managing pain and restoring HRQoL. However, rapid withdrawal of these sources of happiness significantly diminished the level of corticosterone and led to an increase in hyperalgesia in sickle mice. This dramatic depletion of corticosterone following the withdrawal period suggests a displeasure-induced impairment in psychological and/or metabolic states^93^. Therefore, withdrawal of a happy state may lead to worsening pain and inability to cope with distress.

We also performed analysis for depression and anxiety-like behaviors and did not observe any significant difference between AA and SS BERK mice from 3- to 8-months of age on 6 different tests, namely, (i) Elevated Plus Maze, (ii) Forced Swim Test, (iii) Tail Suspension Test, (iv) Novelty-induced Hypophagia, (v) Sucrose Preference Test, and (vi) Stress-induced Hyperthermia upon repeating 3 times with 4 different groups of male mice. All testing was performed in a double-blind manner with highly experienced experts at the Behavioral Phenotyping Core of the National Institute of Neurodegenerative Disorders and Stroke (NINDS) Center at the University of Minnesota. We are further continuing the longitudinal analysis on > 10 month-old male SS and AA BERK mice but high morbidity in male SS mice has delayed obtaining the large numbers of mice. Therefore, these data are not included herein*.*

Besides observing the influence of enriched diet and companionship in transmission and perception of pain in CNS, we also wanted to examine their effect on the pathology of peripheral tissues. We did not observe a significant difference in the pathology of different organs under different treatments (Fig. S2). High protein/calorie diets have been shown to reduce organ injury and improve survival in sickle mice^94^. The difference in our observations could be due to irreversible organ damage in the older sickle mice we used. Increased circulating cytokines have been demonstrated in major depressive disorders and animal models of depression^95–97^. Increased proliferation and mobilization of immune cells, including bone-marrow derived monocytes in the bloodstream, is stimulated by chronic stress^98,99^. Social stress has been shown to compromise blood–brain barrier (BBB) integrity^100^. Activation of mast cells as well as 5-HT in the brain have been associated with increased BBB permeability under stress^101,102^. As a result, circulating cytokines and peripheral immune cells can diffuse into the brain under stress-induced conditions, promoting anxiety and depression^97,103^. Therefore, improved diet and companionship may attenuate chronic hyperalgesia via increased descending modulation from the RVM, and may also show protective effects on peripheral tissues by reducing stress-induced inflammatory response. This is particularly important for SCD, which is associated with depression, anxiety, cognitive impairment, “cytokine storm”, and increased bone-marrow derived hematopoietic and myeloid cells in the circulation^104–106^. Notably, circulating IL6 is highly elevated in sickle patients and sickle mice^107^. It has been shown that social stress stimulates IL6 diffusion into the brain, which acts on the nucleus accumbens resulting in depressive behaviors^100^. In a condition like SCD with a highly inflammatory microenvironment in the wake of a VOC, social stress may promote a vicious cycle of peripheral and central inflammation, depression, anxiety and cognitive impairment, all of which can potentiate pain perception via inhibition of 5-HT releasing mechanism.

Our data suggest that an enhanced psychosocial environment through improved nutrition and companionship attenuates chronic hyperalgesia via increased descending modulation from the RVM involving 5-HT and DA. The observation of increased 5-HT in the SD/C+ group is an important finding of this study. It suggests that pain modulation induced by the descending pathways is mediated by the 5-HT activating system. Therefore, creating a happy environment with alternative and complementary strategies could improve response to analgesic therapies^108–112^. It is likely that the often life-long nature of pain, frequent hospitalization, socio-psychological stress and social stigma in SCD negatively modulates affective mechanisms and interferes with pain management, necessitating high doses of opioids^18^. Arginine supplementation showed a trend towards a decrease in VOC pain and a significant decrease in opioid use in children with SCD^113^. L-glutamine and ω-3 fatty acids also show promising outcomes in reducing VOC and hospital admissions in patients with SCD^114^. Specifically, ω-3 are known for their neuroprotective properties, potentially contributing to protection from sickle cell pathobiology^115^. Ready-to-use supplementary food (RUSF) has been shown to promote growth and improve hematological parameters with no influence on inflammation^116^. The sickle diet contained increased arginine, ω-3 fatty acids and glutamate, compared to the regular diet. The observations on the effect of sickle diet on reducing pain are complemented by our recent observations on the improvement in survival of pups upto 5 months of age of sickle mice fed sickle diet compared to regular diet^117^. In this study, feeding sickle diet to the parents significantly improved the survival of male offsprings, suggesting the vulnerability of male survival in utero and post-natally and sensitivity to the environmental changes. Therefore, some of the effects of diet may be due to an improved metabolic state in SCD.

In conclusion, we show that analgesia is mediated by the 5-HT releasing system acting in the descending pain pathway. Since opioids also work partly on this system, these results suggest that a serotonergic enhancement strategy could be a substitute for opioid use for treating chronic pain. Chronic treatment with opioids leads to addiction, dose escalation, and reduced efficacy due to development of tolerance^8,105^. Enhancement of 5-HT activating system either by psychosocial enrichment or by pharmacological manipulation could induce similar levels of analgesia without causing addiction and tolerance associated with opioid treatment. Additionally, dietary improvement and companionship led to increased analgesic effectiveness of a sub-optimal dose of morphine.

## Materials and methods

### Study design

This study aimed to modulate the psychosocial environment of male sickle mice and examined its effect on hyperalgesia. The psychosocial environment was modulated by providing companionship with a female, enriched diet, and 5-HT regulators either independently or in combination as described below in brief and/or in the accompanying supplementary information. After behavioral evaluation, molecular analyses of 5-HT, DA and corticosterone were performed in tissues collected from male mice exposed to different diets and/or absence/presence of a female companion as described in the “Study groups” below and in the “Results” section.

### Animal handling and procedures

All procedures were approved by the Institutional Animal Care and Use Committee of the University of Minnesota and were conducted in accordance with the statutes of the Animal Welfare Act and the guidelines of the Public Health Service as issued in the Guide for the Care and Use of Laboratory Animals. We used male and female transgenic BERK sickle mice expressing human sickle hemoglobin (HbSS-BERK) and control mice expressing normal human hemoglobin A (HbAA-BERK). Homozygous HbSS-BERK sickle mice with knockout of murine α and β globins carry transgenes for human α and β^S^ globins, and express > 99% human hemoglobin S^53^. These mice feature a severe disease pathology that resembles human sickle cell anemia (SCA), involving hemolysis, reticulocytosis, anemia, extensive organ damage, shortened life span and pain^53,118^. Control HbAA-BERK mice express human α and β^A^ globins exclusively without murine α and β globins. Mice were genotyped for the knockout of mouse globins and presence of human globins (Transnetyx, Cordova, TN) and phenotyped by isoelectric focusing for the presence of homozygous HbS or HbA^118^. The age of both sickle and control mice was between 6 and 10 months.

### Study groups

Both sickle and control mice were divided randomly into the following four study groups based on whether they received the standard rodent, RD, or the sickle diet, SD, and whether they were housed with (C+) or without (C−) a companion of opposite sex. The groups were as follows: RD/C− mice received the standard diet without a companion after birth and throughout the duration of the study; RD/C+ received the standard diet with a companion for the study period after originally being on RD/C− after birth; SD/C− received sickle diet without a companion for the study period after originally being on RD/C− after birth; SD/C+ received both the sickle diet and a companion after birth and for the duration of the study. Three additional study groups examined the impact of withdrawal of the sickle diet, companionship or both. All the withdrawal groups used mice that were treated with both the sickle diet and a companion after birth up to the beginning of the study period, so the baseline reflects the SD/C+ treatment. The withdrawal groups were as follows: SS W SD/C− mice were withdrawn from companionship for the study period after originally being on SD/C+ from birth; SS W RD/C+ mice were withdrawn from the sickle diet for the study period after originally being on SD/C+ from birth; SS W RD/C− mice were withdrawn from both the sickle diet and companionship for the study period after originally being on SD/C+ from birth.

### Diet composition

The RD was a fixed formula regular rodent diet (Teklad Global 18% Protein Rodent Diet) obtained from Teklad Diets (Madison, WI, USA). The SD was a modified version of the Standard Mouse Diet 9F obtained from LabDiet (St.Louis, MO, USA) to better meet the nutritional requirements of sickle mice^119^. The SD is enriched in protein (26.4% vs 18.6%) and fat content (11.1% vs 6.2%). SD also has greater amounts of minerals (potassium, magnesium, iron, zinc copper, and iodine), vitamins (vitamin A, D-3, niacin, folic acid, B-12 and choline chlorine), amino acids (including arginine, and glutamic acid) and omega­3 fatty acid content compared to RD. Mice were fed ad libitum with their respective diets.

### Pharmacological agents

Duloxetine (Dr. Reddy’s Laboratory Ltd, India), a 5-HT-NE reuptake inhibitor, was dissolved in distilled water and administered intraperitoneally to male and female HbAA-BERK and HbSS-BERK mice in the RD/C− treatment group. A single dose at 3, 10, and 15 mg/kg was injected prior to behavioral testing to assess acute analgesic effect^120^. A dose of 3 mg/kg/day of duloxetine was administered to HbSS-BERK mice in the RD/C− treatment group for nine consecutive days to assess the analgesic effect and tolerance behavior over a long-term treatment period.

PCPA (Sigma Aldrich, St. Louis, MO, USA), a 5-HT depletion agent, was prepared and administered as previously described^42^. HbSS-BERK mice in the RD/C− and SD/C+ treatment groups received 100 mg/kg of PCPA each day for 3 days immediately following behavioral testing. A final behavioral assessment was conducted one week after discontinuation of the last PCPA administration.

Morphine sulfate (West-Ward, Eatontown, NJ, USA) dissolved in normal mouse saline at 10 mg/ml was injected subcutaneously to HbSS-BERK mice in the RD/C− and SD/C+ treatment groups. Mice in the RD/C− treatment group were administered either a suboptimal dose of 10 mg/kg or a regular dose of 20 mg/kg. Mice in the SD/C+ treatment group were administered the suboptimal dose of 10 mg/kg. Following injection with morphine, all mice were subjected to behavioral testing to investigate the effect of diet and companionship on opioid analgesia.

### Behavioral testing

Mice were acclimated to handling and testing protocols in a quiet room at controlled temperature of 26–27 °C before being tested for mechanical, thermal (heat and cold), and musculoskeletal hyperalgesia (grip force)^118^. A minimum of 5 min was given between each test to prevent carry-over hyperalgesia.

Mechanical Hyperalgesia: The paw withdrawal frequency (PWF), evoked by 10 applications of a 1.0 g von Frey monofilament (Stoelting Co., Wood Dale, IL, USA) to the plantar surface of each hind paw for one to two seconds with a five second inter-stimulus interval, was measured to determine mechanical sensitivity.

Thermal Hyperalgesia: For heat sensitivity, a stimulus generated by a radiant bulb was applied to the plantar surface of the hind paw, and paw withdrawal latency (PWL), to the nearest 0.1 s, was recorded once the mouse withdrew its paw in response to the stimulus. For cold sensitivity, the PWF on a 4 °C cold plate (Stoelting Co., Wood Dale, IL, USA) over a period of 2 min was determined.

Grip Force: Musculoskeletal hyperalgesia was assessed by peak forepaw grip to a computerized grip force meter (SA Maier Co., Milwaukee, WI, USA). Mice were made to pull on a wire-mesh gauge with their forepaws. The peak force exerted in grams (g) was recorded.

### 5-HT and DA analysis by HPLC

Spinal cords were harvested following euthanasia, swiftly frozen, and stored at a temperature of − 80 °C prior to sample preparation. Frozen spinal cords were homogenized with 0.5 ml of 0.2 M perchloric acid and incubated at 4 °C for 30 min. The homogenates were centrifuged at 20,000*g* for 15 min at 4 °C, and the supernatant was collected and adjusted to pH 3.5 by using 1 M sodium acetate, and then filtered using a 3000 NMWL centrifugal system (Merck Millipore, Billerica, MA, USA). The filtrate was analyzed on an Eicompak SC-3ODS column (ID 3.0 × 100 mm) with AC-ODS Precolumn packing material. The mobile phase was composed of 80% 0.1 M citrate-acetate buffer (pH 3.5) containing 220 mg/l sodium octane sulfonate, 5 mg/l EDTA and 20% (v/v) methanol. A graphite electrode (WE-3G, Gasket GS-25) served as the working electrode and was set at + 750 mv (Eicom) versus an Ag/AGCL reference electrode. The flow rate was set at 340–400 μl/min.

### 5-HT and DA analysis by immunostaining

A brain block containing midbrain and brain stem was sectioned coronally at 6 µm by cryostat to collect the PAG and RVM. Landmarks of the PAG and RVM were identified based on the Allen mouse brain atlas^121^. Cervical spinal cord, PAG, and RVM at 6 µm thickness were labelled with rabbit anti-DA (Abcam, Cambridge, MA, USA) and rat anti-5-HT (Santa Cruz, Dallas, Texas). Cy2-conjugated donkey-anti-rabbit and Cy3-conjugated donkey-anti-rat secondary antibodies (Jackson ImmunoResearch, West Grove, PA, USA) were used to detect immuno-reactivity, and samples were mounted with Vectashield H-1000 (Vector Labs, Burlingame, CA, USA). Images were captured using Olympus IX70 inverted microscope (Olympus Corporation, Center Valley, PA) under 60X objective. The total area of fluorescence corresponding to the labeled regions was measured using Image J (NIH). Data was collected and expressed as total area of fluorescent pixels as described previously^122^.

### Corticosterone analysis by ELISA

Whole blood was collected by cardiac puncture into Eppendorf tubes (Eppendorf North America, Hauppauge, NY). Blood was clotted for 30 min at room temperature before centrifuging for 10 min at 2000 × g. Serum samples were collected and analyzed using the Corticosterone ELISA kit (R&D systems, Minneapolis, MN). Assay results were collected and calculated using the Synergy HT plate reader and Gen5™ 1.0 data analysis software (BioTek).

### Statistical analysis

Analyses reported in Figs. 2 and 3 used one-way repeated-measures analysis of variance (ANOVA) with the Bonferroni post-hoc test and were implemented using Prism (v 7.0c, GraphPad Prism Inc., San Diego, CA). For analyses reported in Figs. 1, 4, 5, and 6, a three-way ANOVA was performed, but no significant three-way interactions were present. Thus, mixed linear models were used correcting for multiple comparisons using Tukey’s post-hoc test. These analyses were done using the lme4 package (v. 1.1-21) in the R system (v. 3.4.0).

The datasets generated during and/or analyzed during the current study are available from the corresponding author on reasonable request.

## Supplementary Information

Supplementary Information 1.

Supplementary Information 2.
