# Supplementary material for: Diet and companionship modulate pain via a serotonergic mechanism
Source: Sci Rep. 2021 Feb 1;11:2330. doi: 10.1038/s41598-021-81654-1 (PMC7851147; doi:10.1038/s41598-021-81654-1)
Supplement: Supplementary file 1 — Supplementary Information 1. [file 41598_2021_81654_MOESM1_ESM.docx]

Diet and companionship modulate pain via a serotonergic mechanism

**Authors:**

Huy Tran^1^, Varun Sagi^1^, Sarita Jarrett^2^, Elise F. Palzer^3^, Rajendra D. Badgaiyan^4^, and Kalpna Gupta^1,5^

^†^*HT and VS have contributed equally to this manuscript.*

**Affiliations:**

^1^Vascular Biology Center, Division of Hematology, Oncology and Transplantation, Department of Medicine, University of Minnesota, Minneapolis, MN, USA.

^2^Northwestern University, Evanston, IL, USA.

^3^Biostatistical Design and Analysis Center, Clinical and Translational Sciences Institute, University of Minnesota, Minneapolis, MN, USA.

^4^Department of Psychiatry, Long School of Medicine, University of Texas Health Science Center, San Antonio, Texas, USA

^5^Hematology/Oncology, Department of Medicine, University of California, Irvine and Southern California Institute for Research and Education, VA Medical Center, Long Beach, CA, USA.

**Supplementary Methods**

**Pathological analysis**

Spleen, liver, and kidney samples obtained following euthanasia were weighed and fixed in formalin, embedded in paraffin, and analyzed in a double-blind manner following staining with H&E and Prussian Blue on all organs, and Periodic Acid-Schiff stain (PAS) only in the kidneys. Tissue histology was reviewed under light microscopy for significant abnormalities. Necrosis, iron levels, nephropathy, and extramedullary hematopoiesis were evaluated at four levels by a board certified veterinary pathologist. The samples were graded objectively on a scale of 0 (absent, not observed) to 4 (marked, severe).

**Supplementary Figures**


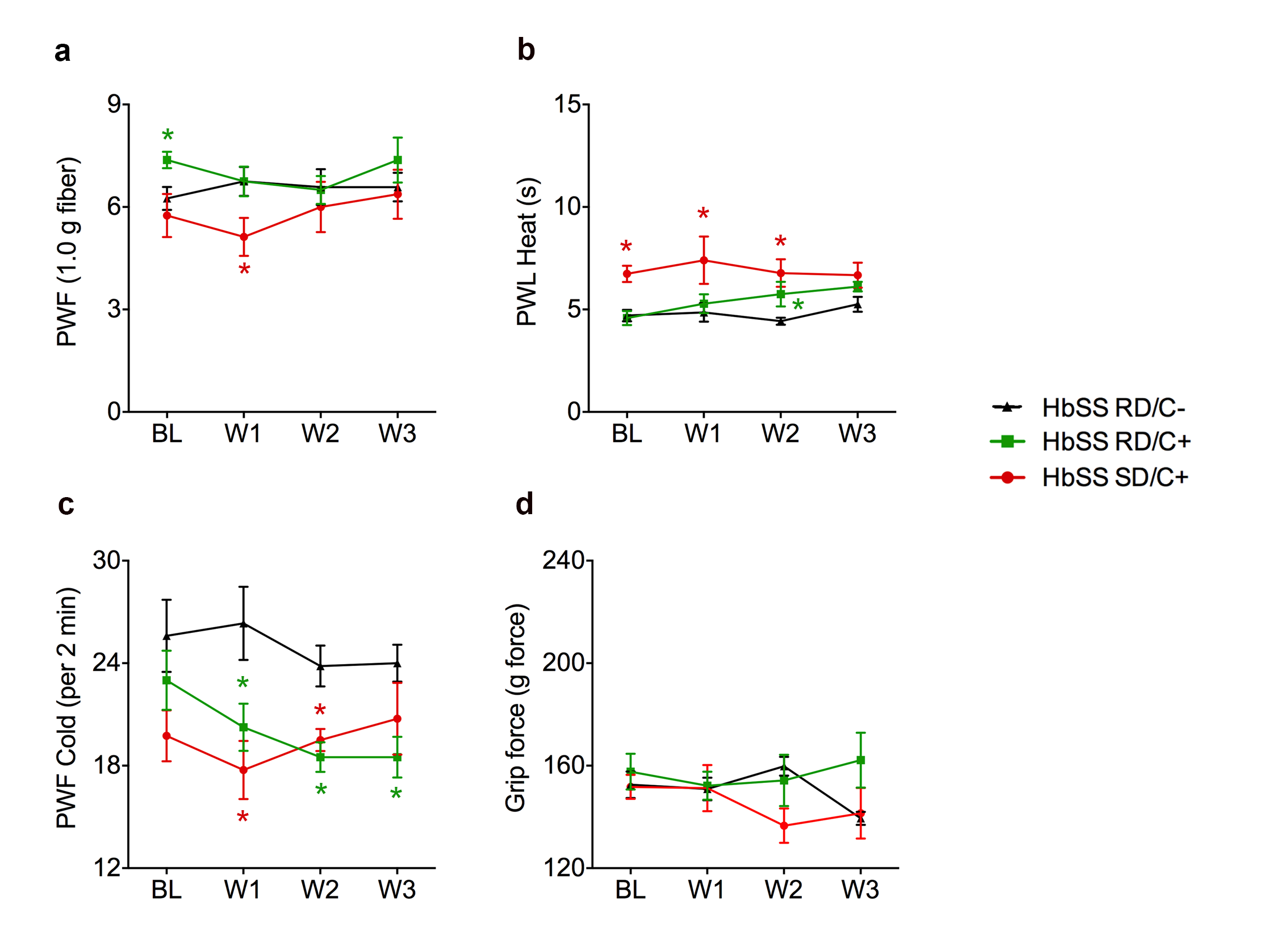


**Figure S1.** *Effect of enriched diet and companionship on hyperalgesia in female sickle mice*. A. PWF in response to 1.0 g von Frey filaments. B. PWL in response to a heat stimulus. C. PWF per 2 min on a cold plate at 4˚C. D. Grip force exerted by forelimbs. Female HbSS-BERK mice between 8-10 months of age were used. Regular diet without companionship, RD/C-, n=6; regular diet with companionship, RD/C+, n=4, sickle diet with companionship, SD/C+, n=4; Unpaired Student’s *t* test, *p<0.05 compared to RD/C-. W1, W2 and W3, weeks 1, 2 and 3, respectively; PWF, paw withdrawal frequency; PWL, paw withdrawal latency.


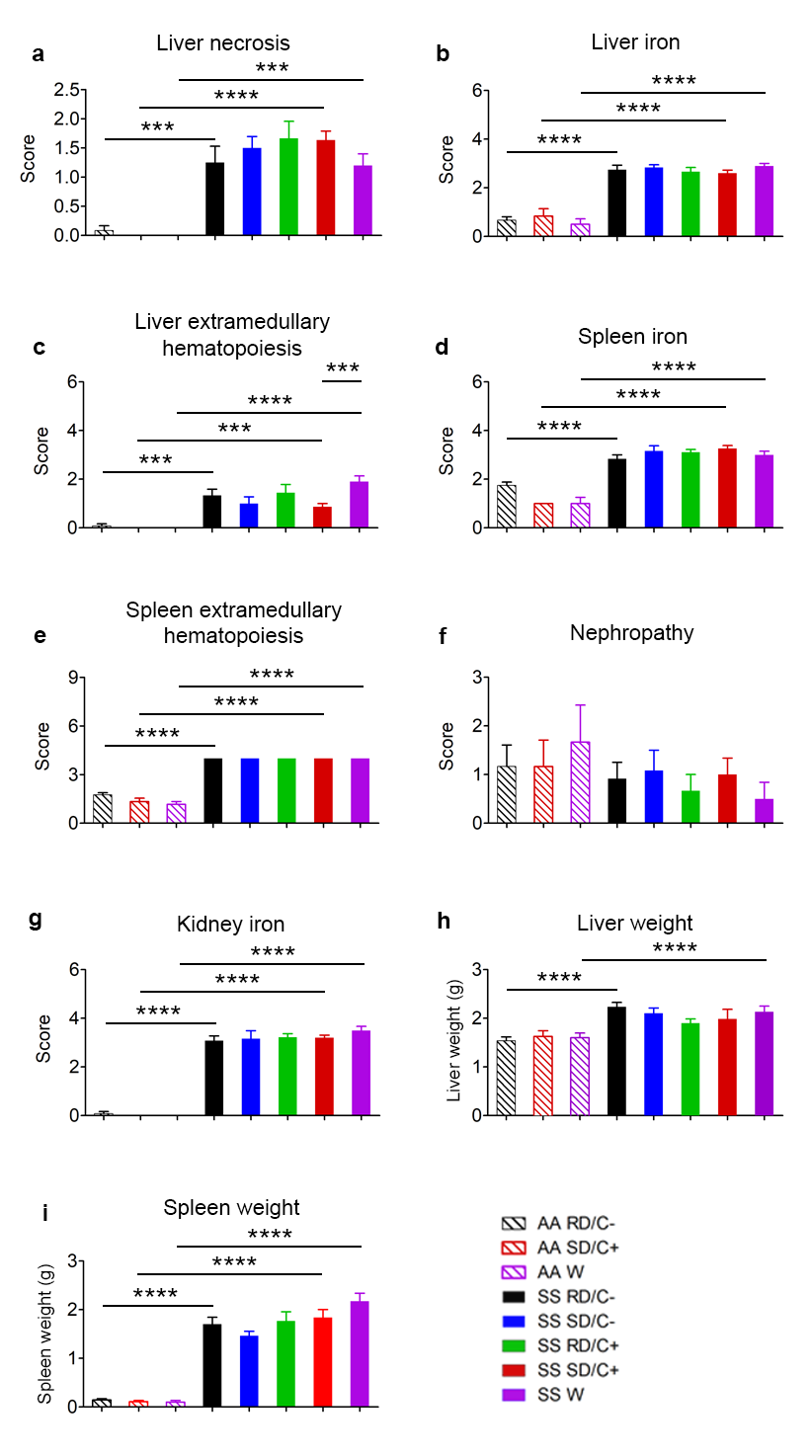


**Figure S2.** *Effect of companionship and enriched diet on organ pathology in male sickle mice.*

A-C. Liver pathology score including necrosis (A), iron deposit (B), and extramedullary hematopoiesis (C). D-E. Spleen pathology score including iron deposit (D) and extramedullary hematopoiesis (E). F-G. Kidney pathology score including nephropathy (F) and iron deposit (G). H. Liver weight. I. Spleen weight. Male HbSS-BERK and HbAA-BERK mice between 8-10 months of age were used. Regular diet without companionship, RD/C-, nSS=12, nAA=12; sickle diet with companionship, SD/C+, nSS=15, nAA=6; withdrawal from sickle diet and companionship, W, nSS=10, nAA=6; sickle diet without companionship, SD/C-, nSS=12; regular diet with companionship, RD/C+, nSS=9. Statistical analysis: Unpaired Student’s *t* test, ***p<0.001, ****p<0.0001.
