# Supplementary material for: Diet and companionship modulate pain via a serotonergic mechanism
Source: Sci Rep. 2021 Feb 1;11:2330. doi: 10.1038/s41598-021-81654-1 (PMC7851147; doi:10.1038/s41598-021-81654-1)
Supplement: Supplementary file 2 — Supplementary Information 2. [file 41598_2021_81654_MOESM2_ESM.pdf]

Diet and companionship modulate pain via a serotonergic mechanism

Authors:
Huy Tran[1], Varun Sagi[1], Sarita Jarrett[2], Elise F. Palzer[3], Rajendra D. Badgaiyan[4], and Kalpna Gupta[1,5]

[†]*HT and VS have contributed equally to this manuscript.*

Affiliations:
[1]Vascular Biology Center, Division of Hematology, Oncology and Transplantation, Department of Medicine, University of Minnesota, Minneapolis, MN, USA.
[2]Northwestern University, Evanston, IL, USA.
[3]Biostatistical Design and Analysis Center, Clinical and Translational Sciences Institute, University of Minnesota, Minneapolis, MN, USA.
[4]Department of Psychiatry, Long School of Medicine, University of Texas Health Science Center, San Antonio, Texas, USA
[5]Hematology/Oncology, Department of Medicine, University of California, Irvine and Southern California Institute for Research and Education, VA Medical Center, Long Beach, CA, USA.

Supplementary Methods

Pathological analysis

Spleen, liver, and kidney samples obtained following euthanasia were weighed and fixed in formalin, embedded in paraffin, and analyzed in a double-blind manner following staining with H&E and Prussian Blue on all organs, and Periodic Acid-Schiff stain (PAS) only in the kidneys. Tissue histology was reviewed under light microscopy for significant abnormalities. Necrosis, iron levels, nephropathy, and extramedullary hematopoiesis were evaluated at four levels by a board certified veterinary pathologist. The samples were graded objectively on a scale of 0 (absent, not observed) to 4 (marked, severe).

Supplementary Statistical Analysis Tables

Figure 1:

Comparing AA RD/C+, AA RD/C-, AA SD/C+, AA RD/C-

Model 1a: PWF(VF)

Linear mixed effects model with random intercept for animal ID and fixed effects for diet, companion, and time with all possible interactions. Data restricted to the 4 AA groups excluding the W groups. Results show that group has a significant effect on PWF (VF)

	Df	Sum Sq	Mean Sq	F value	Pval
group	3	2.164	0.721	32.493	0.138
time	3	0.785	0.262	100.627	0.546
group:time	9	3.472	0.386	99.424	0.405

Model 1b: PWF(VF)

Post hoc analysis of Group effect

Results to all pairwise contrasts of group. Adjusted p-values are calculated using Tukey's Method. Results show that PWF (VF) is significantly higher in AA RD/C- when compared to AA RD/C+ or AA SD/C+ averaging across time.

Contrast	Estimate	Lower CI	Upper CI	Unadjusted P-value	Ajusted P-value
AA RD/C- - AA RD/C+	0.420	0.036	0.804	0.033	0.138
AA RD/C- - AA SD/C-	0.245	-0.139	0.629	0.204	0.574
AA RD/C- - AA SD/C+	0.383	0.007	0.760	0.046	0.185
AA RD/C+ - AA SD/C-	-0.175	-0.528	0.178	0.320	0.743
AA RD/C+ - AA SD/C+	-0.037	-0.382	0.308	0.829	0.996
AA SD/C- - AA SD/C+	0.138	-0.207	0.483	0.420	0.845

Model 2a: PWL Heat(s)

Linear mixed effects model with random intercept for animal ID and fixed effects for diet, companion, and time with all possible interactions. Data restricted to the 4 AA groups excluding the W groups. Results show that group has a significant effect on PWL heat(s)

	Df	Sum Sq	Mean Sq	F value	Pval
group	3	3.672	1.224	23.193	0.232
time	3	19.742	6.581	67.678	$<$0.001
group:time	9	9.768	1.085	67.659	0.224

Model 2b: PWL Heat(s)

Post hoc analysis of Group effect

Results to all pairwise contrasts of group. Adjusted p-values are calculated using Tukey's Method. Results show that AA RD/C- have significantly lower PWL Heat(s) than AA SD/C- averaging across time.

Contrast	Estimate	Lower CI	Upper CI	Unadjusted P-value	Ajusted P-value
AA RD/C- - AA RD/C+	-0.938	-2.160	0.283	0.126	0.404
AA RD/C- - AA SD/C-	-1.157	-2.378	0.064	0.062	0.232
AA RD/C- - AA SD/C+	-0.907	-1.989	0.174	0.096	0.330
AA RD/C+ - AA SD/C-	-0.218	-1.437	1.000	0.714	0.982
AA RD/C+ - AA SD/C+	0.031	-1.047	1.109	0.953	1.000
AA SD/C- - AA SD/C+	0.249	-0.829	1.328	0.637	0.963

Model 3a: PWF Cold

Linear mixed effects model with random intercept for animal ID and fixed effects for diet, companion, and time with all possible interactions. Data restricted to the 4 AA groups excluding the W groups. Results show that group has a significant effect on PWF cold

	Df	Sum Sq	Mean Sq	F value	Pval
group	3	385.577	128.526	37.489	<0.001
time	3	26.860	8.953	106.868	0.011
group:time	9	32.402	3.600	106.156	0.132

Model 3b: PWF Cold

Post hoc analysis of Group effect

Results to all pairwise contrasts of group. Adjusted p-values are calculated using Tukey's Method. Results show that AA RD/C+ and AA SD/C- have significantly higher PWF Cold than AA RD/C- or AA SD/C+ averaging across time.

Contrast	Estimate	Lower CI	Upper CI	Unadjusted P-value	Ajusted P-value
AA RD/C- - AA RD/C+	-3.616	-4.462	-2.770	<0.001	<0.001
AA RD/C- - AA SD/C-	-3.766	-4.612	-2.920	<0.001	<0.001
AA RD/C- - AA SD/C+	-0.152	-0.982	0.678	0.713	0.982
AA RD/C+ - AA SD/C-	-0.150	-0.927	0.627	0.697	0.979
AA RD/C+ - AA SD/C+	3.464	2.705	4.223	<0.001	<0.001
AA SD/C- - AA SD/C+	3.614	2.855	4.373	<0.001	<0.001

Model 4a: Grip force

Linear mixed effects model with random intercept for animal ID and fixed effects for diet, companion, and time with all possible interactions. Data restricted to the 4 AA groups excluding the W groups. Results show that group has a significant effect on Grip force.

	Df	Sum Sq	Mean Sq	F value	Pval
group	3	1099.620	366.540	34.011	0.040
time	3	1318.960	439.653	101.137	0.014
group:time	9	833.759	92.640	101.142	0.632

Model 4b: Grip Force

Post hoc analysis of Group effect

Results to all pairwise contrasts of group. Adjusted p-values are calculated using Tukey's Method. Results show that AA RD/C+ and AA SD/C- have significantly lower Grip Force than AA RD/C- or AA SD/C+ averaging across time.

Contrast	Estimate	Lower CI	Upper CI	Unadjusted P-value	Ajusted P-value
AA RD/C- - AA RD/C+	11.869	1.355	22.384	0.028	0.119
AA RD/C- - AA SD/C-	11.875	1.653	22.097	0.024	0.104
AA RD/C- - AA SD/C+	1.234	-9.635	12.103	0.819	0.996
AA RD/C+ - AA SD/C-	0.005	-10.729	10.740	0.999	1.000
AA RD/C+ - AA SD/C+	-10.636	-21.988	0.716	0.065	0.245
AA SD/C- - AA SD/C+	-10.641	-21.723	0.440	0.059	0.226

Comparing SS RD/C+, SS RD/C-, SS SD/C+, SS RD/C-

Model 5a: PWF (VF)

Linear mixed effects model with random intercept for animal ID and fixed effects for diet, companion, and time with all possible interactions. Data restricted to the 4 SS groups excluding the Withdrawal groups.

	Df	Sum Sq	Mean Sq	F value	Pval
group	3	46.782	15.594	51.105	<0.001
time	3	54.553	18.184	142.075	<0.001
group:time	9	45.337	5.037	142.096	<0.001

Model 5b: PWF (VF)

Post Hoc analysis of Group effect

Results to contrasts of the main effect. Adjusted p-values are calculated using Tukey's Method. Results show that SS RD/C- have significantly higher PWF (VF) than all 3 other groups. Similarly, SS SD/C+ was found to have significantly lower PWF (VF) than all 3 other groups.

Contrast	Estimate	Lower CI	Upper CI	Unadjusted P-value	Ajusted P-value
SS RD/C- - SS RD/C+	1.714	1.025	2.403	<0.001	<0.001
SS RD/C- - SS SD/C-	1.590	0.941	2.239	<0.001	<0.001
SS RD/C- - SS SD/C+	2.891	2.300	3.483	<0.001	<0.001
SS RD/C+ - SS SD/C-	-0.124	-0.799	0.551	0.713	0.983
SS RD/C+ - SS SD/C+	1.178	0.557	1.798	<0.001	0.002
SS SD/C- - SS SD/C+	1.302	0.727	1.877	<0.001	<0.001

Model 5c: PWF (VF)

Post Hoc analysis of Group Time interaction effect at Baseline

Results to contrasts of of group at baseline. Adjusted p-values are calculated using Tukey's Method. Results show that SS SD/C+ have significantly lower PWF (VF) values at baseline compared to each of the other groups.

Contrast	Time	Estimate	Lower CI	Upper CI	Unadjusted P-value	Ajusted P-value
SS RD/C- - SS RD/C+	BL	0.042	-0.803	0.888	0.921	1
SS RD/C- - SS SD/C-	BL	-0.041	-0.832	0.750	0.918	1
SS RD/C- - SS SD/C+	BL	2.730	2.009	3.451	<0.001	<0.001
SS RD/C+ - SS SD/C-	BL	-0.083	-0.914	0.747	0.843	1
SS RD/C+ - SS SD/C+	BL	2.687	1.923	3.452	<0.001	<0.001
SS SD/C- - SS SD/C+	BL	2.771	2.067	3.474	<0.001	<0.001

Model 5d: PWF (VF)

Post Hoc analysis of Group Time interaction effect at week 3

Results to contrasts of group at week 3. Adjusted p-values are calculated using Tukey's Method. Results show that SS RD/C- have significantly higher PWF (VF) values at 3 weeks compared to each of the other groups.

	Contrast	Time	Estimate	Lower CI	Upper CI	Unadjusted P-value	Ajusted P-value
19	SS RD/C- - SS RD/C+	W3	2.314	1.444	3.184	<0.001	<0.001
20	SS RD/C- - SS SD/C-	W3	2.240	1.413	3.067	<0.001	<0.001
21	SS RD/C- - SS SD/C+	W3	2.683	1.920	3.447	<0.001	<0.001
22	SS RD/C+ - SS SD/C-	W3	-0.074	-0.914	0.766	0.862	1
23	SS RD/C+ - SS SD/C+	W3	0.370	-0.409	1.148	0.349	0.935
24	SS SD/C- - SS SD/C+	W3	0.443	-0.286	1.173	0.231	0.834

Model 6a: PWL Heat(s)

Linear mixed effects model with random intercept for animal ID and fixed effects for diet, companion, and time with all possible interactions. Data restricted to the 4 SS groups excluding the Withdrawal groups.

	Df	Sum Sq	Mean Sq	F value	Pval
group	3	82.881	27.627	50.902	<0.001
time	3	77.247	25.749	144.497	<0.001
group:time	9	90.678	10.075	144.537	<0.001

Model 6b: PWL Heat(s)

Post Hoc analysis of Group effect

Results to contrasts of the main effect. Adjusted p-values are calculated using Tukey's Method. Results show that SS RD/C+ and SS SD/C+ have significantly higher PWL Heat(s) than either of the two other groups averaged over time.

Contrast	Estimate	Lower CI	Upper CI	Unadjusted P-value	Ajusted P-value
SS RD/C- - SS RD/C+	-2.619	-3.271	-1.966	<0.001	<0.001
SS RD/C- - SS SD/C-	-1.634	-2.250	-1.018	<0.001	<0.001
SS RD/C- - SS SD/C+	-2.884	-3.444	-2.323	<0.001	<0.001
SS RD/C+ - SS SD/C-	0.985	0.345	1.625	0.003	0.017
SS RD/C+ - SS SD/C+	-0.265	-0.852	0.322	0.369	0.801
SS SD/C- - SS SD/C+	-1.250	-1.796	-0.704	<0.001	<0.001

Model 6c: PWL Heat(s)

Post Hoc analysis of Group Time interaction effect at Baseline

Results to contrasts of of group at baseline. Adjusted p-values are calculated using Tukey's Method. Results show that SS SD/C+ have significantly higher PWL Heat(s) values at baseline compared to each of the other groups.

Contrast	Time	Estimate	Lower CI	Upper CI	Unadjusted P-value	Ajusted P-value
SS RD/C- - SS RD/C+	BL	-0.019	-0.904	0.866	0.966	1
SS RD/C- - SS SD/C-	BL	0.206	-0.621	1.033	0.623	0.996
SS RD/C- - SS SD/C+	BL	-2.595	-3.349	-1.840	<0.001	<0.001
SS RD/C+ - SS SD/C-	BL	0.225	-0.644	1.094	0.61	0.996
SS RD/C+ - SS SD/C+	BL	-2.576	-3.376	-1.776	<0.001	<0.001
SS SD/C- - SS SD/C+	BL	-2.801	-3.537	-2.065	<0.001	<0.001

Model 6d: PWL Heat(s)

Post Hoc analysis of Group Time interaction effect at week 3

Results to contrasts of group at week 3. Adjusted p-values are calculated using Tukey's Method. Results show that SS RD/C- have significantly lower PWL Heat(s) values at 3 weeks compared to each of the other groups.

	Contrast	Time	Estimate	Lower CI	Upper CI	Unadjusted P-value	Ajusted P-value
19	SS RD/C- - SS RD/C+	W3	-3.690	-4.591	-2.790	<0.001	<0.001
20	SS RD/C- - SS SD/C-	W3	-2.720	-3.577	-1.863	<0.001	<0.001
21	SS RD/C- - SS SD/C+	W3	-2.807	-3.599	-2.016	<0.001	<0.001
22	SS RD/C+ - SS SD/C-	W3	0.970	0.089	1.852	0.031	0.256
23	SS RD/C+ - SS SD/C+	W3	0.883	0.065	1.701	0.035	0.277
24	SS SD/C- - SS SD/C+	W3	-0.087	-0.858	0.683	0.823	1

Model 7a: PWF Cold

Linear mixed effects model with random intercept for animal ID and fixed effects for diet, companion, and time with all possible interactions. Data restricted to the 4 SS groups excluding the Withdrawal groups.

	Df	Sum Sq	Mean Sq	F value	Pval
group	3	120.957	40.319	49.599	<0.001
time	3	262.639	87.546	142.864	<0.001
group:time	9	212.405	23.601	142.862	<0.001

Model 7b: PWF Cold

Post Hoc analysis of Group effect

Results to contrasts of the main effect. Adjusted p-values are calculated using Tukey's Method. Results show that SS RD/C- have significantly higher PWF Cold values compared to each of the other groups averaged over time.

Contrast	Estimate	Lower CI	Upper CI	Unadjusted P-value	Ajusted P-value
SS RD/C- - SS RD/C+	2.727	1.180	4.275	<0.001	0.005
SS RD/C- - SS SD/C-	3.821	2.330	5.313	<0.001	<0.001
SS RD/C- - SS SD/C+	3.497	2.165	4.829	<0.001	<0.001
SS RD/C+ - SS SD/C-	1.094	-0.435	2.624	0.157	0.483
SS RD/C+ - SS SD/C+	0.769	-0.605	2.144	0.266	0.676
SS SD/C- - SS SD/C+	-0.325	-1.636	0.986	0.621	0.959

Model 7c: PWF Cold

Post Hoc analysis of Group Time interaction effect at Baseline

Results to contrasts of of group at baseline. Adjusted p-values are calculated using Tukey's Method. Results show that SS SD/C+ have significantly lower PWF Cold values compared to each of the other groups at baseline.

Contrast	Time	Estimate	Lower CI	Upper CI	Unadjusted P-value	Ajusted P-value
SS RD/C- - SS RD/C+	BL	-0.436	-2.506	1.633	0.677	0.998
SS RD/C- - SS SD/C-	BL	0.947	-1.030	2.924	0.345	0.933
SS RD/C- - SS SD/C+	BL	4.164	2.386	5.941	<0.001	<0.001
SS RD/C+ - SS SD/C-	BL	1.383	-0.644	3.411	0.179	0.757
SS RD/C+ - SS SD/C+	BL	4.600	2.766	6.434	<0.001	<0.001
SS SD/C- - SS SD/C+	BL	3.217	1.487	4.946	<0.001	0.004

Model 7d: PWF Cold

Post Hoc analysis of Group Time interaction effect at week 3

Results to contrasts of group at week 3. Adjusted p-values are calculated using Tukey's Method. Results show that SS RD/C- have significantly higher PWF Cold values compared to each of the other groups at week 3.

	Contrast	Time	Estimate	Lower CI	Upper CI	Unadjusted P-value	Ajusted P-value
19	SS RD/C- - SS RD/C+	W3	3.973	1.904	6.042	<0.001	0.003
20	SS RD/C- - SS SD/C-	W3	4.920	2.911	6.930	<0.001	<0.001
21	SS RD/C- - SS SD/C+	W3	3.014	1.224	4.804	0.001	0.014
22	SS RD/C+ - SS SD/C-	W3	0.948	-1.112	3.008	0.364	0.943
23	SS RD/C+ - SS SD/C+	W3	-0.959	-2.805	0.888	0.306	0.908
24	SS SD/C- - SS SD/C+	W3	-1.907	-3.686	-0.127	0.036	0.284

Model 8a: Grip Force

Linear mixed effects model with random intercept for animal ID and fixed effects for diet, companion, and time with all possible interactions. Data restricted to the 4 SS groups excluding the Withdrawal groups.

	Df	Sum Sq	Mean Sq	F value	Pval
group	3	3209.131	1069.710	50.282	0.040
time	3	222.377	74.126	146.928	0.014
group:time	9	1348.796	149.866	146.934	0.632

Model 8b: Grip Force

Post Hoc analysis of Group effect

Results to contrasts of the main effect. Adjusted p-values are calculated using Tukey's Method. Results show that SS SD/C+ have significantly higher grip force values compared to each of the other groups averaged over time.

Contrast	Estimate	Lower CI	Upper CI	Unadjusted P-value	Ajusted P-value
SS RD/C- - SS RD/C+	-7.476	-18.422	3.470	0.176	0.523
SS RD/C- - SS SD/C-	-5.304	-15.589	4.980	0.305	0.730
SS RD/C- - SS SD/C+	-18.880	-28.261	-9.499	<0.001	0.001
SS RD/C+ - SS SD/C-	2.172	-8.526	12.870	0.685	0.977
SS RD/C+ - SS SD/C+	-11.404	-21.237	-1.571	0.024	0.105
SS SD/C- - SS SD/C+	-13.576	-22.667	-4.484	0.004	0.021

Model 8c: Grip Force

Post Hoc analysis of Group Time interaction effect at Baseline

Results to contrasts of of group at baseline. Adjusted p-values are calculated using Tukey's Method. Results show that SS SD/C+ have significantly higher grip force values compared to each of the other groups at baseline.

Contrast	Time	Estimate	Lower CI	Upper CI	Unadjusted P-value	Ajusted P-value
SS RD/C- - SS RD/C+	BL	-1.315	-15.609	12.978	0.856	1.000
SS RD/C- - SS SD/C-	BL	-2.128	-15.491	11.236	0.753	1.000
SS RD/C- - SS SD/C+	BL	-19.760	-31.950	-7.571	0.002	0.020
SS RD/C+ - SS SD/C-	BL	-0.813	-14.854	13.229	0.909	1.000
SS RD/C+ - SS SD/C+	BL	-18.445	-31.374	-5.516	0.006	0.060
SS SD/C- - SS SD/C+	BL	-17.632	-29.525	-5.739	0.004	0.045

Model 8d: Grip Force

Post Hoc analysis of Group Time interaction effect at week 3

Results to contrasts of group at week 3. Adjusted p-values are calculated using Tukey's Method. No significant results were found at week 3

	Contrast	Time	Estimate	Lower CI	Upper CI	Unadjusted P-value	Ajusted P-value
19	SS RD/C- - SS RD/C+	W3	-6.067	-20.600	8.467	0.410	0.962
20	SS RD/C- - SS SD/C-	W3	1.888	-11.927	15.703	0.787	1.000
21	SS RD/C- - SS SD/C+	W3	-12.293	-24.851	0.265	0.055	0.385
22	SS RD/C+ - SS SD/C-	W3	7.955	-6.275	22.185	0.271	0.878
23	SS RD/C+ - SS SD/C+	W3	-6.226	-19.239	6.787	0.346	0.934
24	SS SD/C- - SS SD/C+	W3	-14.181	-26.386	-1.976	0.023	0.202

Comparing SS W RD/C+, SS W SD/C-, and SS W RD/C- to SS SD/C+

Model 9a: PWF (VF)

Linear mixed effects model with random intercept for animal ID and fixed effects for diet, companion, and time with all possible interactions. Data restricted to the 4 SS groups excluding the Withdrawal groups.

	Df	Sum Sq	Mean Sq	F value	Pval
group	3	37.079	12.360	46.677	<0.001
time	3	33.435	11.145	133.217	<0.001
group:time	9	55.366	6.152	132.978	<0.001

Model 9b: PWF (VF)

Post Hoc analysis of Group effect

Results to contrasts of the main effect. Adjusted p-values are calculated using Tukey's Method. Results show that SS W RD/C- and SS W SD/C- have significantly higher PWF (VF) than either of the two other groups averaged over time.

Contrast	Estimate	Lower CI	Upper CI	Unadjusted P-value	Ajusted P-value
SS SD/C+ - SS W RD/C-	-1.452	-1.904	-1.001	<0.001	<0.001
SS SD/C+ - SS W RD/C+	0.234	-0.492	0.960	0.52	0.916
SS SD/C+ - SS W SD/C-	-1.690	-2.401	-0.979	<0.001	<0.001
SS W RD/C- - SS W RD/C+	1.687	0.962	2.411	<0.001	<0.001
SS W RD/C- - SS W SD/C-	-0.237	-0.946	0.471	0.503	0.906
SS W RD/C+ - SS W SD/C-	-1.924	-2.833	-1.015	<0.001	<0.001

Model 9c: PWF (VF)

Post Hoc analysis of Group Time interaction effect at baseline

Results to contrasts of group at baseline. Adjusted p-values are calculated using Tukey's Method. No significant results were found at baseline.

Contrast	Time	Estimate	Lower CI	Upper CI	Unadjusted P-value	Ajusted P-value
SS SD/C+ - SS W RD/C-	BL	-0.130	-0.742	0.483	0.676	0.998
SS SD/C+ - SS W RD/C+	BL	0.396	-0.572	1.364	0.420	0.965
SS SD/C+ - SS W SD/C-	BL	0.596	-0.372	1.564	0.226	0.828
SS W RD/C- - SS W RD/C+	BL	0.526	-0.443	1.494	0.285	0.891
SS W RD/C- - SS W SD/C-	BL	0.726	-0.243	1.694	0.141	0.676
SS W RD/C+ - SS W SD/C-	BL	0.200	-1.025	1.425	0.747	1.000

Model 9d: PWF (VF)

Post Hoc analysis of Group Time interaction effect at week 3

Results to contrasts of group at week 3. Adjusted p-values are calculated using Tukey's Method. Results show that SS W RD/C- and SS W SD/C- have significantly higher PWF (VF) than either of the two other groups at week 3.

	Contrast	Time	Estimate	Lower CI	Upper CI	Unadjusted P-value	Ajusted P-value
19	SS SD/C+ - SS W RD/C-	W3	-2.185	-2.818	-1.552	<0.001	<0.001
20	SS SD/C+ - SS W RD/C+	W3	0.175	-0.880	1.230	0.743	0.999
21	SS SD/C+ - SS W SD/C-	W3	-3.160	-4.142	-2.179	<0.001	<0.001
22	SS W RD/C- - SS W RD/C+	W3	2.360	1.317	3.403	<0.001	<0.001
23	SS W RD/C- - SS W SD/C-	W3	-0.975	-1.943	-0.007	0.048	0.352
24	SS W RD/C+ - SS W SD/C-	W3	-3.335	-4.620	-2.051	<0.001	<0.001

Model 10a: PWL Heat(s)

Linear mixed effects model with random intercept for animal ID and fixed effects for diet, companion, and time with all possible interactions. Data restricted to the 4 SS groups excluding the Withdrawal groups.

	Df	Sum Sq	Mean Sq	F value	Pval
group	3	35.215	11.738	46.600	<0.001
time	3	26.961	8.987	127.043	<0.001
group:time	9	67.586	7.510	126.933	<0.001

Model 10b: PWL Heat(s)

Post Hoc analysis of Group effect

Results to contrasts of the main effect. Adjusted p-values are calculated using Tukey's Method. Results show that SS W SD/C- have significantly lower PWL Heat(s) than the each of the other 3 groups averaged over time.

Contrast	Estimate	Lower CI	Upper CI	Unadjusted P-value	Ajusted P-value
SS SD/C+ - SS W RD/C-	0.560	-0.047	1.167	0.07	0.261
SS SD/C+ - SS W RD/C+	-0.899	-1.857	0.058	0.065	0.246
SS SD/C+ - SS W SD/C-	2.070	1.197	2.943	<0.001	<0.001
SS W RD/C- - SS W RD/C+	-1.459	-2.422	-0.496	0.004	0.019
SS W RD/C- - SS W SD/C-	1.510	0.631	2.389	0.001	0.006
SS W RD/C+ - SS W SD/C-	2.969	1.820	4.118	<0.001	<0.001

Model 10c: PWL Heat(s)

Post Hoc analysis of Group Time interaction effect at baseline

Results to contrasts of group at baseline. Adjusted p-values are calculated using Tukey's Method. Results show that SS W RD/C- have significantly higher PWL Heat(s) than the each of the other 3 groups at baseline.

Contrast	Time	Estimate	Lower CI	Upper CI	Unadjusted P-value	Ajusted P-value
SS SD/C+ - SS W RD/C-	BL	-1.028	-1.836	-0.221	0.013	0.126
SS SD/C+ - SS W RD/C+	BL	-0.112	-1.389	1.165	0.862	1.000
SS SD/C+ - SS W SD/C-	BL	0.039	-1.150	1.227	0.949	1.000
SS W RD/C- - SS W RD/C+	BL	0.916	-0.360	2.193	0.158	0.715
SS W RD/C- - SS W SD/C-	BL	1.067	-0.122	2.256	0.078	0.485
SS W RD/C+ - SS W SD/C-	BL	0.151	-1.395	1.697	0.847	1.000

Model 10d: PWL Heat(s)

Post Hoc analysis of Group Time interaction effect at week 3

Results to contrasts of group at week 3. Adjusted p-values are calculated using Tukey's Method. Results show that SS W RD/C- have significantly lower PWL Heat(s) than the each of the other 3 groups at week 3.

	Contrast	Time	Estimate	Lower CI	Upper CI	Unadjusted P-value	Ajusted P-value
19	SS SD/C+ - SS W RD/C-	W3	1.783	0.810	2.756	<0.001	0.005
20	SS SD/C+ - SS W RD/C+	W3	-0.994	-2.386	0.397	0.16	0.72
21	SS SD/C+ - SS W SD/C-	W3	3.174	1.967	4.381	<0.001	<0.001
22	SS W RD/C- - SS W RD/C+	W3	-2.777	-4.241	-1.313	<0.001	0.003
23	SS W RD/C- - SS W SD/C-	W3	1.391	0.102	2.681	0.035	0.277
24	SS W RD/C+ - SS W SD/C-	W3	4.168	2.540	5.797	<0.001	<0.001

Model 11a: PWF Cold

Linear mixed effects model with random intercept for animal ID and fixed effects for diet, companion, and time with all possible interactions. Data restricted to the 4 SS groups excluding the Withdrawal groups.

	Df	Sum Sq	Mean Sq	F value	Pval
group	3	150.297	50.099	47.954	<0.001
time	3	207.980	69.327	139.170	<0.001
group:time	9	223.594	24.844	138.694	<0.001

Model 11b: PWF Cold

Post Hoc analysis of Group effect

Results to contrasts of the main effect. Adjusted p-values are calculated using Tukey's Method. Results show that SS W RD/C- have significantly higher PWF Cold than the each of the other 3 groups averaged over time.

Contrast	Estimate	Lower CI	Upper CI	Unadjusted P-value	Ajusted P-value
SS SD/C+ - SS W RD/C-	-1.394	-2.627	-0.161	0.027	0.119
SS SD/C+ - SS W RD/C+	0.453	-1.533	2.440	0.649	0.968
SS SD/C+ - SS W SD/C-	-4.943	-6.754	-3.131	<0.001	<0.001
SS W RD/C- - SS W RD/C+	1.848	-0.137	3.832	0.067	0.254
SS W RD/C- - SS W SD/C-	-3.548	-5.358	-1.739	<0.001	0.001
SS W RD/C+ - SS W SD/C-	-5.396	-7.784	-3.008	<0.001	<0.001

Model 11c: PWF Cold

Post Hoc analysis of Group Time interaction effect at baseline

Results to contrasts of group at baseline. Adjusted p-values are calculated using Tukey's Method. Results show that SS W RD/C- have significantly higher PWF Cold than the each of the other 3 groups at baseline.

Contrast	Time	Estimate	Lower CI	Upper CI	Unadjusted P-value	Ajusted P-value
SS SD/C+ - SS W RD/C-	BL	0.650	-1.015	2.315	0.441	0.972
SS SD/C+ - SS W RD/C+	BL	1.224	-1.408	3.856	0.359	0.941
SS SD/C+ - SS W SD/C-	BL	0.007	-2.444	2.457	0.996	1.000
SS W RD/C- - SS W RD/C+	BL	0.574	-2.058	3.206	0.667	0.998
SS W RD/C- - SS W SD/C-	BL	-0.643	-3.094	1.807	0.604	0.995
SS W RD/C+ - SS W SD/C-	BL	-1.217	-4.405	1.970	0.451	0.974

Model 11d: PWF Cold

Post Hoc analysis of Group Time interaction effect at week 3

Results to contrasts of group at week 3. Adjusted p-values are calculated using Tukey's Method. Results show that SS W RD/C- have significantly higher PWF Cold than the each of the other 3 groups at week 3.

	Contrast	Time	Estimate	Lower CI	Upper CI	Unadjusted P-value	Ajusted P-value
19	SS SD/C+ - SS W RD/C-	W3	-2.591	-4.273	-0.909	0.003	0.033
20	SS SD/C+ - SS W RD/C+	W3	-0.418	-3.261	2.424	0.772	1
21	SS SD/C+ - SS W SD/C-	W3	-7.983	-10.445	-5.521	<0.001	<0.001
22	SS W RD/C- - SS W RD/C+	W3	2.173	-0.660	5.005	0.132	0.655
23	SS W RD/C- - SS W SD/C-	W3	-5.392	-7.842	-2.941	<0.001	<0.001
24	SS W RD/C+ - SS W SD/C-	W3	-7.564	-10.919	-4.210	<0.001	<0.001

Model 12a: Grip Force

Linear mixed effects model with random intercept for animal ID and fixed effects for diet, companion, and time with all possible interactions. Data restricted to the 4 SS groups excluding the Withdrawal groups.

	Df	Sum Sq	Mean Sq	F value	Pval
group	3	1821.374	607.125	47.403	0.120
time	3	38.983	12.994	138.607	0.988
group:time	9	2703.146	300.350	138.135	0.433

Model 12b: Grip Force

Post Hoc analysis of Group effect

Results to contrasts of the main effect. Adjusted p-values are calculated using Tukey's Method. Results show that SS SD/C+ have significantly higher grip force than SS W Rd/C+ and SS W SD/C- averaged over time.

Contrast	Estimate	Lower CI	Upper CI	Unadjusted P-value	Ajusted P-value
SS SD/C+ - SS W RD/C-	6.272	-3.948	16.491	0.223	0.608
SS SD/C+ - SS W RD/C+	-13.321	-29.782	3.139	0.110	0.374
SS SD/C+ - SS W SD/C-	-1.171	-16.192	13.850	0.876	0.999
SS W RD/C- - SS W RD/C+	-19.593	-36.037	-3.150	0.021	0.091
SS W RD/C- - SS W SD/C-	-7.443	-22.445	7.559	0.323	0.751
SS W RD/C+ - SS W SD/C-	12.151	-7.638	31.939	0.223	0.608

Model 12c: Grip Force

Post Hoc analysis of Group Time interaction effect at baseline

Results to contrasts of group at baseline. Adjusted p-values are calculated using Tukey's Method. Results show that SS W RD/C+ and SS W SD/C- have significantly lower grip force than either of the two other groups at baseline.

Contrast	Time	Estimate	Lower CI	Upper CI	Unadjusted P-value	Ajusted P-value
SS SD/C+ - SS W RD/C-	BL	0.166	-13.525	13.857	0.981	1.000
SS SD/C+ - SS W RD/C+	BL	-2.558	-24.206	19.089	0.815	1.000
SS SD/C+ - SS W SD/C-	BL	5.124	-15.029	25.276	0.616	0.996
SS W RD/C- - SS W RD/C+	BL	-2.724	-24.372	18.924	0.804	1.000
SS W RD/C- - SS W SD/C-	BL	4.958	-15.195	25.111	0.627	0.997
SS W RD/C+ - SS W SD/C-	BL	7.682	-18.535	33.899	0.563	0.992

Model 12d: Grip Force

Post Hoc analysis of Group Time interaction effect at week 3

Results to contrasts of group at week 3. Adjusted p-values are calculated using Tukey's Method. No significant results were found at week 3.

	Contrast	Time	Estimate	Lower CI	Upper CI	Unadjusted P-value	Ajusted P-value
19	SS SD/C+ - SS W RD/C-	W3	5.763	-8.068	19.593	0.411	0.963
20	SS SD/C+ - SS W RD/C+	W3	-25.313	-48.668	-1.958	0.034	0.272
21	SS SD/C+ - SS W SD/C-	W3	-6.139	-26.387	14.108	0.550	0.991
22	SS W RD/C- - SS W RD/C+	W3	-31.076	-54.348	-7.803	0.009	0.095
23	SS W RD/C- - SS W SD/C-	W3	-11.902	-32.055	8.251	0.245	0.851
24	SS W RD/C+ - SS W SD/C-	W3	19.174	-8.399	46.746	0.171	0.742

Figure 4:

Compare Vehicle, 3mg/kg Duloxetine, 10 mg/kg Dulox-etine

Model 1a: Males - PWF (VF)

	Df	Sum Sq	Mean Sq	F value	Pval
group	2	0.099	0.049	21.021	0.863
EN_time	7	2.015	0.288	146.043	0.536
group:EN_time	14	8.044	0.575	146.042	0.056

Model 1b: Males - PWF (VF)

Post Hoc analysis of Group Time interaction effect at 0.5 hours

Results to contrasts of of group at 0.5 hours. Adjusted p-values are calculated using Tukey's Method.

	Contrast	Time	Estimate	Lower CI	Upper CI	Unadjusted P-value	Ajusted P-value
10	10 mg/kg - 3 mg/kg	30 mins	0.875	-0.033	1.783	0.058	0.139
11	10 mg/kg - Vehicle	30 mins	0.438	-0.470	1.345	0.337	0.600
12	3 mg/kg - Vehicle	30 mins	-0.437	-1.345	0.470	0.337	0.600

Model 1c: Males - PWF (VF)

Post Hoc analysis of Group Time interaction effect at 8 hours

Results to contrasts of group at 8 hours. Adjusted p-values are calculated using Tukey's Method.

	Contrast	Time	Estimate	Lower CI	Upper CI	Unadjusted P-value	Ajusted P-value
19	10 mg/kg - 3 mg/kg	8 hr	0.329	-0.592	1.250	0.477	0.755
20	10 mg/kg - Vehicle	8 hr	0.329	-0.592	1.250	0.477	0.755
21	3 mg/kg - Vehicle	8 hr	0.000	-0.908	0.908	1.000	1.000

Model 2a: Males - PWL Heat(s)

	Df	Sum Sq	Mean Sq	F value	Pval
group	2	30.382	15.191	21.111	0.002
EN_time	7	330.849	47.264	144.208	<0.001
group:EN_time	14	205.613	14.687	144.187	<0.001

Model 2b: Males - PWL Heat(s)

Post Hoc analysis of Group Time interaction effect at 0.5 hours

Results to contrasts of of group at 0.5 hours. Adjusted p-values are calculated using Tukey's Method.

	Contrast	Time	Estimate	Lower CI	Upper CI	Unadjusted P-value	Ajusted P-value
10	10 mg/kg - 3 mg/kg	30 mins	1.406	-0.312	3.123	0.107	0.24
11	10 mg/kg - Vehicle	30 mins	5.498	3.781	7.216	<0.001	<0.001
12	3 mg/kg - Vehicle	30 mins	4.093	2.375	5.810	<0.001	<0.001

Model 2c: Males - PWL Heat(s)

Post Hoc analysis of Group Time interaction effect at 8 hours

Results to contrasts of group at 8 hours. Adjusted p-values are calculated using Tukey's Method.

	Contrast	Time	Estimate	Lower CI	Upper CI	Unadjusted P-value	Ajusted P-value
19	10 mg/kg - 3 mg/kg	8 hr	0.386	-1.488	2.261	0.683	0.912
20	10 mg/kg - Vehicle	8 hr	0.376	-1.498	2.250	0.691	0.916
21	3 mg/kg - Vehicle	8 hr	-0.010	-1.728	1.707	0.990	1.000

Model 3a: Males - PWF Cold

	Df	Sum Sq	Mean Sq	F value	Pval
group	2	282.631	141.316	21.171	<0.001
EN_time	7	1967.124	281.018	144.311	<0.001
group:EN_time	14	917.217	65.515	144.280	<0.001

Model 3b: Males - PWF Cold

Post Hoc analysis of Group Time interaction effect at 0.5 hours

Results to contrasts of of group at 0.5 hours. Adjusted p-values are calculated using Tukey's Method.

	Contrast	Time	Estimate	Lower CI	Upper CI	Unadjusted P-value	Ajusted P-value
10	10 mg/kg - 3 mg/kg	30 mins	-1.375	-4.380	1.630	0.366	0.637
11	10 mg/kg - Vehicle	30 mins	-11.750	-14.755	-8.745	<0.001	<0.001
12	3 mg/kg - Vehicle	30 mins	-10.375	-13.380	-7.370	<0.001	<0.001

Model 3c: Males - PWF Cold

Post Hoc analysis of Group Time interaction effect at 8 hours

Results to contrasts of group at 8 hours. Adjusted p-values are calculated using Tukey's Method.

	Contrast	Time	Estimate	Lower CI	Upper CI	Unadjusted P-value	Ajusted P-value
19	10 mg/kg - 3 mg/kg	8 hr	-0.512	-3.840	2.816	0.761	0.950
20	10 mg/kg - Vehicle	8 hr	-1.262	-4.590	2.066	0.454	0.734
21	3 mg/kg - Vehicle	8 hr	-0.750	-3.755	2.255	0.622	0.874

Model 4a: Males - Grip Force

	Df	Sum Sq	Mean Sq	F value	Pval
group	2	55.734	27.867	20.956	0.714
EN_time	7	5255.848	750.835	143.975	<0.001
group:EN_time	14	3019.715	215.694	143.971	0.002

Model 4b: Males - Grip Force

Post Hoc analysis of Group Time interaction effect at 0.5 hours

Results to contrasts of of group at 0.5 hours. Adjusted p-values are calculated using Tukey's Method.

	Contrast	Time	Estimate	Lower CI	Upper CI	Unadjusted P-value	Ajusted P-value
10	10 mg/kg - 3 mg/kg	30 mins	-16.500	-37.145	4.145	0.113	0.248
11	10 mg/kg - Vehicle	30 mins	2.875	-17.770	23.520	0.778	0.957
12	3 mg/kg - Vehicle	30 mins	19.375	-1.270	40.020	0.065	0.152

Model 4c: Males - Grip Force

Post Hoc analysis of Group Time interaction effect at 8 hours

Results to contrasts of group at 8 hours. Adjusted p-values are calculated using Tukey's Method.

	Contrast	Time	Estimate	Lower CI	Upper CI	Unadjusted P-value	Ajusted P-value
19	10 mg/kg - 3 mg/kg	8 hr	-8.768	-30.001	12.465	0.407	0.682
20	10 mg/kg - Vehicle	8 hr	-5.810	-27.042	15.423	0.582	0.844
21	3 mg/kg - Vehicle	8 hr	2.958	-17.687	23.603	0.772	0.954

Model 5a: Females - PWF (VF)

	Df	Sum Sq	Mean Sq	F value	Pval
group	2	0.648	0.324	17	0.265
EN_time	7	2.351	0.336	119	0.177
group:EN_time	14	3.891	0.278	119	0.260

Model 5b: Females - PWF (VF)

Post Hoc analysis of Group Time interaction effect at 0.5 hours

Results to contrasts of of group at 0.5 hours. Adjusted p-values are calculated using Tukey's Method.

	Contrast	Time	Estimate	Lower CI	Upper CI	Unadjusted P-value	Ajusted P-value
10	10 mg/kg - 3 mg/kg	30 mins	0.187	-0.652	1.027	0.655	0.895
11	10 mg/kg - Vehicle	30 mins	0.437	-0.402	1.277	0.300	0.550
12	3 mg/kg - Vehicle	30 mins	0.250	-0.435	0.935	0.467	0.745

Model 5c: Females - PWF (VF)

Post Hoc analysis of Group Time interaction effect at 8 hours

Results to contrasts of group at 8 hours. Adjusted p-values are calculated using Tukey's Method.

	Contrast	Time	Estimate	Lower CI	Upper CI	Unadjusted P-value	Ajusted P-value
19	10 mg/kg - 3 mg/kg	8 hr	0.750	-0.089	1.589	0.079	0.181
20	10 mg/kg - Vehicle	8 hr	0.375	-0.464	1.214	0.373	0.644
21	3 mg/kg - Vehicle	8 hr	-0.375	-1.060	0.310	0.277	0.518

Model 6a: Females - PWL Heat(s)

	Df	Sum Sq	Mean Sq	F value	Pval
group	2	60.418	30.209	17	<0.001
EN_time	7	800.716	114.388	119	<0.001
group:EN_time	14	451.307	32.236	119	<0.001

Model 6b: Females - PWL Heat(s)

Post Hoc analysis of Group Time interaction effect at 0.5 hours

Results to contrasts of of group at 0.5 hours. Adjusted p-values are calculated using Tukey's Method.

	Contrast	Time	Estimate	Lower CI	Upper CI	Unadjusted P-value	Ajusted P-value
10	10 mg/kg - 3 mg/kg	30 mins	3.302	0.868	5.737	0.009	0.024
11	10 mg/kg - Vehicle	30 mins	10.444	8.009	12.878	<0.001	<0.001
12	3 mg/kg - Vehicle	30 mins	7.141	5.154	9.129	<0.001	<0.001

Model 6c: Females - PWL Heat(s)

Post Hoc analysis of Group Time interaction effect at 8 hours

Results to contrasts of group at 8 hours. Adjusted p-values are calculated using Tukey's Method.

	Contrast	Time	Estimate	Lower CI	Upper CI	Unadjusted P-value	Ajusted P-value
19	10 mg/kg - 3 mg/kg	8 hr	-0.383	-2.817	2.052	0.753	0.946
20	10 mg/kg - Vehicle	8 hr	0.163	-2.271	2.598	0.893	0.990
21	3 mg/kg - Vehicle	8 hr	0.546	-1.442	2.534	0.582	0.845

Model 7a: Females - PWF Cold

	Df	Sum Sq	Mean Sq	F value	Pval
group	2	67.348	33.674	17	0.02
EN_time	7	756.059	108.008	119	<0.001
group:EN_time	14	498.641	35.617	119	<0.001

Model 7b: Females - PWF Cold

Post Hoc analysis of Group Time interaction effect at 0.5 hours

Results to contrasts of of group at 0.5 hours. Adjusted p-values are calculated using Tukey's Method.

	Contrast	Time	Estimate	Lower CI	Upper CI	Unadjusted P-value	Ajusted P-value
10	10 mg/kg - 3 mg/kg	30 mins	4.375	-0.209	8.959	0.061	0.144
11	10 mg/kg - Vehicle	30 mins	-4.500	-9.084	0.084	0.054	0.13
12	3 mg/kg - Vehicle	30 mins	-8.875	-12.618	-5.132	<0.001	<0.001

Model 7c: Females - PWF Cold

Post Hoc analysis of Group Time interaction effect at 8 hours

Results to contrasts of group at 8 hours. Adjusted p-values are calculated using Tukey's Method.

	Contrast	Time	Estimate	Lower CI	Upper CI	Unadjusted P-value	Ajusted P-value
19	10 mg/kg - 3 mg/kg	8 hr	0.375	-4.209	4.959	0.870	0.985
20	10 mg/kg - Vehicle	8 hr	-1.500	-6.084	3.084	0.514	0.789
21	3 mg/kg - Vehicle	8 hr	-1.875	-5.618	1.868	0.319	0.576

Model 8a: Females - Grip Force

	Df	Sum Sq	Mean Sq	F value	Pval
group	2	14.017	7.009	17.014	0.876
EN_time	7	603.209	86.173	118.995	0.131
group:EN_time	14	1418.651	101.332	118.995	0.030

Model 8b: Females - Grip Force

Post Hoc analysis of Group Time interaction effect at 0.5 hours

Results to contrasts of of group at 0.5 hours. Adjusted p-values are calculated using Tukey's Method.

	Contrast	Time	Estimate	Lower CI	Upper CI	Unadjusted P-value	Ajusted P-value
10	10 mg/kg - 3 mg/kg	30 mins	-11.083	-25.487	3.321	0.128	0.276
11	10 mg/kg - Vehicle	30 mins	-7.417	-21.821	6.988	0.304	0.555
12	3 mg/kg - Vehicle	30 mins	3.667	-8.094	15.428	0.532	0.804

Model 8c: Females - Grip Force

Post Hoc analysis of Group Time interaction effect at 8 hours

Results to contrasts of group at 8 hours. Adjusted p-values are calculated using Tukey's Method.

	Contrast	Time	Estimate	Lower CI	Upper CI	Unadjusted P-value	Ajusted P-value
19	10 mg/kg - 3 mg/kg	8 hr	-12.458	-26.863	1.946	0.088	0.200
20	10 mg/kg - Vehicle	8 hr	-14.229	-28.633	0.175	0.053	0.126
21	3 mg/kg - Vehicle	8 hr	-1.771	-13.532	9.990	0.762	0.950

Figure 4: Vehicle and 3 mg/kg Duloxetine for long term treatment

Model 9a: Males - PWF (VF)

	Df	Sum Sq	Mean Sq	F value	Pval
group	1	0.587	0.587	60	0.306
EN_time	5	1.059	0.212	60	0.857
group:EN_time	5	0.059	0.012	60	1.000

Model 9b: Males - PWF (VF)

Post Hoc analysis of Group Time interaction effect at Day 1

Results to contrasts of of group at day 1. Adjusted p-values are calculated using Tukey's Method.

Contrast	Time	Estimate	Lower CI	Upper CI	Unadjusted P-value	Ajusted P-value
3 mg/kg - Vehicle	1hr D1	-0.167	-1.024	0.69	0.699	0.699

Model 9c: Males - PWF (VF)

Post Hoc analysis of Group Time interaction effect at Day 9

Results to contrasts of group at day 9. Adjusted p-values are calculated using Tukey's Method.

	Contrast	Time	Estimate	Lower CI	Upper CI	Unadjusted P-value	Ajusted P-value
5	3 mg/kg - Vehicle	1hr D9	-0.25	-1.107	0.607	0.562	0.562

Model 10a: Males - PWL Heat(s)

	Df	Sum Sq	Mean Sq	F value	Pval
group	1	44.781	44.781	10	<0.001
EN_time	5	67.656	13.531	50	<0.001
group:EN_time	5	76.160	15.232	50	<0.001

Model 10b: Males - PWL Heat(s)

Post Hoc analysis of Group Time interaction effect at Day 1

Results to contrasts of of group at day 1. Adjusted p-values are calculated using Tukey's Method.

Contrast	Time	Estimate	Lower CI	Upper CI	Unadjusted P-value	Ajusted P-value
3 mg/kg - Vehicle	1hr D1	3.933	2.181	5.686	<0.001	<0.001

Model 10c: Males - PWL Heat(s)

Post Hoc analysis of Group Time interaction effect at Day 9

Results to contrasts of group at day 9. Adjusted p-values are calculated using Tukey's Method.

	Contrast	Time	Estimate	Lower CI	Upper CI	Unadjusted P-value	Ajusted P-value
5	3 mg/kg - Vehicle	1hr D9	3.3	1.548	5.052	<0.001	<0.001

Model 11a: Males - PWF Cold

	Df	Sum Sq	Mean Sq	F value	Pval
group	1	833.681	833.681	60	<0.001
EN_time	5	284.569	56.914	60	<0.001
group:EN_time	5	217.236	43.447	60	<0.001

Model 11b: Males - PWF Cold

Post Hoc analysis of Group Time interaction effect at Day 1

Results to contrasts of of group at day 1. Adjusted p-values are calculated using Tukey's Method.

Contrast	Time	Estimate	Lower CI	Upper CI	Unadjusted P-value	Ajusted P-value
3 mg/kg - Vehicle	1hr D1	-7.667	-9.556	-5.778	<0.001	<0.001

Model 11c: Males - PWF Cold

Post Hoc analysis of Group Time interaction effect at Day 9

Results to contrasts of group at day 9. Adjusted p-values are calculated using Tukey's Method.

	Contrast	Time	Estimate	Lower CI	Upper CI	Unadjusted P-value	Ajusted P-value
5	3 mg/kg - Vehicle	1hr D9	-8.833	-10.722	-6.944	<0.001	<0.001

Model 12a: Males - Grip Force

	Df	Sum Sq	Mean Sq	F value	Pval
group	1	7.560	7.560	10	0.654
EN_time	5	177.292	35.458	50	0.427
group:EN_time	5	106.378	21.276	50	0.700

Model 12b: Males - Grip Force

Post Hoc analysis of Group Time interaction effect at Day 1

Results to contrasts of of group at day 1. Adjusted p-values are calculated using Tukey's Method.

Contrast	Time	Estimate	Lower CI	Upper CI	Unadjusted P-value	Ajusted P-value
3 mg/kg - Vehicle	1hr D1	3.056	-4.48	10.591	0.42	0.42

Model 12c: Males - Grip Force

Post Hoc analysis of Group Time interaction effect at Day 9

Results to contrasts of group at day 9. Adjusted p-values are calculated using Tukey's Method.

	Contrast	Time	Estimate	Lower CI	Upper CI	Unadjusted P-value	Ajusted P-value
5	3 mg/kg - Vehicle	1hr D9	0.278	-7.258	7.813	0.941	0.941

Model 13a: Females - PWF (VF)

	Df	Sum Sq	Mean Sq	F value	Pval
group	1	0.198	0.198	18.965	0.575
EN_time	5	3.291	0.658	93.033	0.373
group:EN_time	5	2.385	0.477	93.033	0.562

Model 13b: Females - PWF (VF)

Post Hoc analysis of Group Time interaction effect at Day 1

Results to contrasts of of group at day 1. Adjusted p-values are calculated using Tukey's Method.

Contrast	Time	Estimate	Lower CI	Upper CI	Unadjusted P-value	Ajusted P-value
3 mg/kg - Vehicle	1hr D1	-0.288	-1.479	0.902	0.626	0.626

Model 13c: Females - PWF (VF)

Post Hoc analysis of Group Time interaction effect at Day 9

Results to contrasts of group at day 9. Adjusted p-values are calculated using Tukey's Method.

	Contrast	Time	Estimate	Lower CI	Upper CI	Unadjusted P-value	Ajusted P-value
5	3 mg/kg - Vehicle	1hr D9	-0.245	-1.455	0.965	0.684	0.684

Model 14a: Females - PWL Heat(s)

	Df	Sum Sq	Mean Sq	F value	Pval
group	1	16.422	16.422	19.086	0.002
EN_time	5	110.718	22.144	93.132	<0.001
group:EN_time	5	87.771	17.554	93.132	<0.001

Model 14b: Females - PWL Heat(s)

Post Hoc analysis of Group Time interaction effect at Day 1

Results to contrasts of of group at day 1. Adjusted p-values are calculated using Tukey's Method.

Contrast	Time	Estimate	Lower CI	Upper CI	Unadjusted P-value	Ajusted P-value
3 mg/kg - Vehicle	1hr D1	3.556	1.49	5.621	0.001	0.001

Contrast	Time	Estimate	Lower CI	Upper CI	Unadjusted P-value	Ajusted P-value

Model 14c: Females - PWL Heat(s)

Post Hoc analysis of Group Time interaction effect at Day 9

Results to contrasts of group at day 9. Adjusted p-values are calculated using Tukey's Method.

	Contrast	Time	Estimate	Lower CI	Upper CI	Unadjusted P-value	Ajusted P-value
5	3 mg/kg - Vehicle	1hr D9	3.638	1.547	5.729	0.001	0.001

Model 15a: Females - PWF Cold

	Df	Sum Sq	Mean Sq	F value	Pval
group	1	659.611	659.611	19.505	<0.001
EN_time	5	1229.468	245.894	93.878	<0.001
group:EN_time	5	195.443	39.089	93.878	0.003

Model 15b: Females - PWF Cold

Post Hoc analysis of Group Time interaction effect at Day 1

Results to contrasts of of group at day 1. Adjusted p-values are calculated using Tukey's Method.

Contrast	Time	Estimate	Lower CI	Upper CI	Unadjusted P-value	Ajusted P-value
3 mg/kg - Vehicle	1hr D1	-7.837	-10.865	-4.808	<0.001	<0.001

Model 15c: Females - PWF Cold

Post Hoc analysis of Group Time interaction effect at Day 9

Results to contrasts of group at day 9. Adjusted p-values are calculated using Tukey's Method.

	Contrast	Time	Estimate	Lower CI	Upper CI	Unadjusted P-value	Ajusted P-value
5	3 mg/kg - Vehicle	1hr D9	-7.946	-11.099	-4.792	<0.001	<0.001

Model 16a: Females - Grip Force

	Df	Sum Sq	Mean Sq	F value	Pval
group	1	813.769	813.769	18.694	0.038
EN_time	5	1675.214	335.043	90.927	0.080
group:EN_time	5	447.546	89.509	90.927	0.741

Model 16b: Females - Grip Force

Post Hoc analysis of Group Time interaction effect at Day 1

Results to contrasts of of group at day 1. Adjusted p-values are calculated using Tukey's Method.

Contrast	Time	Estimate	Lower CI	Upper CI	Unadjusted P-value	Ajusted P-value
3 mg/kg - Vehicle	1hr D1	6.843	-7.073	20.759	0.33	0.33

Model 16c: Females - Grip Force

Post Hoc analysis of Group Time interaction effect at Day 9

Results to contrasts of group at day 9. Adjusted p-values are calculated using Tukey's Method.

	Contrast	Time	Estimate	Lower CI	Upper CI	Unadjusted P-value	Ajusted P-value
5	3 mg/kg - Vehicle	1hr D9	16.147	0.398	31.897	0.045	0.045

Figure 5: Compare RD/C- Veh, RD/C- 100 mg/kg PCPA, SD/C+ 100 mg/kg PCPA

Model 17a: PWF (VF)

	Df	Sum Sq	Mean Sq	F value	Pval
group	2	1.317	0.659	13	0.058
EN_time	4	7.760	1.940	52	<0.001
group:EN_time	8	7.523	0.940	52	<0.001

Model 17b: PWF (VF)

Post Hoc analysis of Group Time interaction effect at baseline

Results to contrasts of of group at baseline. Adjusted p-values are calculated using Tukey's Method.

Contrast	Time	Estimate	Lower CI	Upper CI	Unadjusted P-value	Ajusted P-value
RD/C- PCPA - RD/C- Vehicle	BL	-0.417	-1.080	0.247	0.211	0.42
RD/C- PCPA - SD/C+ PCPA	BL	1.167	0.425	1.909	0.003	0.008
RD/C- Vehicle - SD/C+ PCPA	BL	1.583	0.841	2.325	<0.001	<0.001

Model 17c: PWF (VF)

Post Hoc analysis of Group Time interaction effect at Day 3

Results to contrasts of group at day 3. Adjusted p-values are calculated using Tukey's Method.

	Contrast	Time	Estimate	Lower CI	Upper CI	Unadjusted P-value	Ajusted P-value
13	RD/C- PCPA - RD/C- Vehicle	D3	0.667	0.003	1.330	0.049	0.118
14	RD/C- PCPA - SD/C+ PCPA	D3	0.417	-0.325	1.159	0.263	0.498
15	RD/C- Vehicle - SD/C+ PCPA	D3	-0.250	-0.992	0.492	0.499	0.775

Model 17d: PWF (VF)

Post Hoc analysis of Group Time interaction effect at Day 10

Results to contrasts of group at day 10. Adjusted p-values are calculated using Tukey's Method.

	Contrast	Time	Estimate	Lower CI	Upper CI	Unadjusted P-value	Ajusted P-value
7	RD/C- PCPA - RD/C- Vehicle	D10	-0.417	-1.080	0.247	0.211	0.420
8	RD/C- PCPA - SD/C+ PCPA	D10	0.750	0.008	1.492	0.048	0.115
9	RD/C- Vehicle - SD/C+ PCPA	D10	1.167	0.425	1.909	0.003	0.008

Model 18a: PWL Heat(s)

	Df	Sum Sq	Mean Sq	F value	Pval
group	2	1.677	0.839	13	0.224
EN_time	4	13.795	3.449	52	<0.001
group:EN_time	8	12.208	1.526	52	0.007

Model 18b: PWL Heat(s)

Post Hoc analysis of Group Time interaction effect at baseline

Results to contrasts of of group at baseline. Adjusted p-values are calculated using Tukey's Method.

Contrast	Time	Estimate	Lower CI	Upper CI	Unadjusted P-value	Ajusted P-value
RD/C- PCPA - RD/C- Vehicle	BL	0.172	-0.971	1.315	0.762	0.950
RD/C- PCPA - SD/C+ PCPA	BL	-1.377	-2.655	-0.098	0.036	0.088
RD/C- Vehicle - SD/C+ PCPA	BL	-1.549	-2.827	-0.270	0.019	0.049

Model 18c: PWL Heat(s)

Post Hoc analysis of Group Time interaction effect at Day 3

Results to contrasts of group at day 3. Adjusted p-values are calculated using Tukey's Method.

	Contrast	Time	Estimate	Lower CI	Upper CI	Unadjusted P-value	Ajusted P-value
13	RD/C- PCPA - RD/C- Vehicle	D3	0.137	-1.006	1.281	0.809	0.968
14	RD/C- PCPA - SD/C+ PCPA	D3	0.805	-0.474	2.083	0.209	0.416
15	RD/C- Vehicle - SD/C+ PCPA	D3	0.668	-0.611	1.946	0.296	0.544

Model 18d: PWL Heat(s)

Post Hoc analysis of Group Time interaction effect at Day 10

Results to contrasts of group at day 10. Adjusted p-values are calculated using Tukey's Method.

	Contrast	Time	Estimate	Lower CI	Upper CI	Unadjusted P-value	Ajusted P-value
7	RD/C- PCPA - RD/C- Vehicle	D10	0.262	-0.882	1.405	0.645	0.888
8	RD/C- PCPA - SD/C+ PCPA	D10	-1.680	-2.959	-0.402	0.012	0.030
9	RD/C- Vehicle - SD/C+ PCPA	D10	-1.942	-3.220	-0.663	0.004	0.011

Model 19a: PWF Cold

	Df	Sum Sq	Mean Sq	F value	Pval
group	2	55.009	27.505	13	<0.001
EN_time	4	34.476	8.619	52	0.004
group:EN_time	8	32.583	4.073	52	0.055

Model 19b: PWF Cold

Post Hoc analysis of Group Time interaction effect at baseline

Results to contrasts of of group at baseline. Adjusted p-values are calculated using Tukey's Method.

Contrast	Time	Estimate	Lower CI	Upper CI	Unadjusted P-value	Ajusted P-value
RD/C- PCPA - RD/C- Vehicle	BL	-1	-2.797	0.797	0.27	0.509
RD/C- PCPA - SD/C+ PCPA	BL	4	1.990	6.010	<0.001	<0.001
RD/C- Vehicle - SD/C+ PCPA	BL	5	2.990	7.010	<0.001	<0.001

Model 19c: PWF Cold

Post Hoc analysis of Group Time interaction effect at Day 3

Results to contrasts of group at day 3. Adjusted p-values are calculated using Tukey's Method.

	Contrast	Time	Estimate	Lower CI	Upper CI	Unadjusted P-value	Ajusted P-value
13	RD/C- PCPA - RD/C- Vehicle	D3	1.000	-0.797	2.797	0.270	0.509
14	RD/C- PCPA - SD/C+ PCPA	D3	1.833	-0.176	3.843	0.073	0.170
15	RD/C- Vehicle - SD/C+ PCPA	D3	0.833	-1.176	2.843	0.410	0.686

Model 19d: PWF Cold

Post Hoc analysis of Group Time interaction effect at Day 10

Results to contrasts of group at day 10. Adjusted p-values are calculated using Tukey's Method.

	Contrast	Time	Estimate	Lower CI	Upper CI	Unadjusted P-value	Ajusted P-value
7	RD/C- PCPA - RD/C- Vehicle	D10	-1.167	-2.964	0.631	0.199	0.401
8	RD/C- PCPA - SD/C+ PCPA	D10	2.500	0.490	4.510	0.016	0.041
9	RD/C- Vehicle - SD/C+ PCPA	D10	3.667	1.657	5.676	<0.001	0.002

Model 20a: Grip Force

	Df	Sum Sq	Mean Sq	F value	Pval
group	2	561.365	280.682	13	0.021
EN_time	4	350.097	87.524	52	0.175
group:EN_time	8	2065.056	258.132	52	<0.001

Model 20b: Grip Force

Post Hoc analysis of Group Time interaction effect at baseline

Results to contrasts of of group at baseline. Adjusted p-values are calculated using Tukey's Method.

Contrast	Time	Estimate	Lower CI	Upper CI	Unadjusted P-value	Ajusted P-value
RD/C- PCPA - RD/C- Vehicle	BL	-6.722	-15.919	2.475	0.149	0.316
RD/C- PCPA - SD/C+ PCPA	BL	-21.250	-31.533	-10.967	<0.001	<0.001
RD/C- Vehicle - SD/C+ PCPA	BL	-14.528	-24.810	-4.245	0.006	0.017

Model 20c: Grip Force

Post Hoc analysis of Group Time interaction effect at Day 3

Results to contrasts of group at day 3. Adjusted p-values are calculated using Tukey's Method.

	Contrast	Time	Estimate	Lower CI	Upper CI	Unadjusted P-value	Ajusted P-value
13	RD/C- PCPA - RD/C- Vehicle	D3	-12.500	-21.697	-3.303	0.009	0.023
14	RD/C- PCPA - SD/C+ PCPA	D3	7.556	-2.727	17.838	0.147	0.312
15	RD/C- Vehicle - SD/C+ PCPA	D3	20.056	9.773	30.338	<0.001	<0.001

Model 20d: Grip Force

Post Hoc analysis of Group Time interaction effect at Day 10

Results to contrasts of group at day 10. Adjusted p-values are calculated using Tukey's Method.

	Contrast	Time	Estimate	Lower CI	Upper CI	Unadjusted P-value	Ajusted P-value
7	RD/C- PCPA - RD/C- Vehicle	D10	-8.444	-17.641	0.753	0.071	0.166
8	RD/C- PCPA - SD/C+ PCPA	D10	4.667	-5.616	14.949	0.367	0.637
9	RD/C- Vehicle - SD/C+ PCPA	D10	13.111	2.829	23.394	0.013	0.035

Figure 6: Compare RD/C- vehicle, RD/C- 10 mg/kg morphine, RD/C- 20 mg/kg morphine, and SD/C+ 10 mg/kg morphine

Model 21a: PWF (VF)

	Df	Sum Sq	Mean Sq	F value	Pval
group	3	35.000	11.667	15	<0.001
EN_time	4	294.858	73.714	60	<0.001
group:EN_time	12	116.430	9.703	60	<0.001

Model 21b: PWF (VF)

Post Hoc analysis of Group Time interaction effect at 0.5 hours

Results to contrasts of of group at 0.5 hours. Adjusted p-values are calculated using Tukey's Method.

	Contrast	Estimate	Lower CI	Upper CI	Unadjusted P-value	Ajusted P-value
13	RD/C- 10 mg/kg - RD/C- 20 mg/kg	1.8	0.638	2.962	0.003	0.036
14	RD/C- 10 mg/kg - RD/C- Vehicle	-5.0	-6.096	-3.904	<0.001	<0.001
15	RD/C- 10 mg/kg - SD/C+ 10 mg/kg	1.8	0.704	2.896	0.002	0.022
16	RD/C- 20 mg/kg - RD/C- Vehicle	-6.8	-7.962	-5.638	<0.001	<0.001
17	RD/C- 20 mg/kg - SD/C+ 10 mg/kg	0.0	-1.162	1.162	1	1
18	RD/C- Vehicle - SD/C+ 10 mg/kg	6.8	5.704	7.896	<0.001	<0.001

Model 21c: PWF (VF)

Post Hoc analysis of Group Time interaction effect at 4 hours

Results to contrasts of group at 4 hours. Adjusted p-values are calculated using Tukey's Method.

	Contrast	Estimate	Lower CI	Upper CI	Unadjusted P-value	Ajusted P-value
7	RD/C- 10 mg/kg - RD/C- 20 mg/kg	-0.925	-2.087	0.237	0.003	0.599
8	RD/C- 10 mg/kg - RD/C- Vehicle	-0.300	-1.396	0.796	<0.001	0.993
9	RD/C- 10 mg/kg - SD/C+ 10 mg/kg	1.200	0.104	2.296	0.002	0.255
10	RD/C- 20 mg/kg - RD/C- Vehicle	0.625	-0.537	1.787	0.003	0.884
11	RD/C- 20 mg/kg - SD/C+ 10 mg/kg	2.125	0.963	3.287	<0.001	0.008
12	RD/C- Vehicle - SD/C+ 10 mg/kg	1.500	0.404	2.596	0.002	0.084

Model 22a: PWL Heat(s)

	Df	Sum Sq	Mean Sq	F value	Pval
group	3	74.529	24.843	15	<0.001
EN_time	4	103.412	25.853	60	<0.001
group:EN_time	12	64.610	5.384	60	<0.001

Model 22b: PWL Heat(s)

Post Hoc analysis of Group Time interaction effect at 0.5 hours

Results to contrasts of of group at 0.5 hours. Adjusted p-values are calculated using Tukey's Method.

	Contrast	Estimate	Lower CI	Upper CI	Unadjusted P-value	Ajusted P-value
13	RD/C- 10 mg/kg - RD/C- 20 mg/kg	-3.565	-4.380	-2.750	<0.001	<0.001
14	RD/C- 10 mg/kg - RD/C- Vehicle	1.335	0.567	2.103	<0.001	0.012
15	RD/C- 10 mg/kg - SD/C+ 10 mg/kg	-5.327	-6.095	-4.559	<0.001	<0.001
16	RD/C- 20 mg/kg - RD/C- Vehicle	4.900	4.085	5.715	<0.001	<0.001
17	RD/C- 20 mg/kg - SD/C+ 10 mg/kg	-1.762	-2.577	-0.947	<0.001	<0.001
18	RD/C- Vehicle - SD/C+ 10 mg/kg	-6.662	-7.430	-5.894	<0.001	<0.001

Model 22c: PWL Heat(s)

Post Hoc analysis of Group Time interaction effect at 4 hours

Results to contrasts of group at 4 hours. Adjusted p-values are calculated using Tukey's Method.

	Contrast	Estimate	Lower CI	Upper CI	Unadjusted P-value	Ajusted P-value
7	RD/C- 10 mg/kg - RD/C- 20 mg/kg	-0.469	-1.284	0.346	<0.001	0.858
8	RD/C- 10 mg/kg - RD/C- Vehicle	-0.475	-1.243	0.293	<0.001	0.817
9	RD/C- 10 mg/kg - SD/C+ 10 mg/kg	-2.790	-3.558	-2.022	<0.001	<0.001
10	RD/C- 20 mg/kg - RD/C- Vehicle	-0.006	-0.821	0.809	<0.001	1
11	RD/C- 20 mg/kg - SD/C+ 10 mg/kg	-2.321	-3.136	-1.506	<0.001	<0.001
12	RD/C- Vehicle - SD/C+ 10 mg/kg	-2.315	-3.083	-1.547	<0.001	<0.001

Model 23a: PWF Cold

	Df	Sum Sq	Mean Sq	F value	Pval
group	3	336.391	112.130	15	<0.001
EN_time	4	723.573	180.893	60	<0.001
group:EN_time	12	319.633	26.636	60	<0.001

Model 23b: PWF Cold

Post Hoc analysis of Group Time interaction effect at 0.5 hours

Results to contrasts of of group at 0.5 hours. Adjusted p-values are calculated using Tukey's Method.

	Contrast	Estimate	Lower CI	Upper CI	Unadjusted P-value	Ajusted P-value
13	RD/C- 10 mg/kg - RD/C- 20 mg/kg	3.45	1.356	5.544	0.002	0.019
14	RD/C- 10 mg/kg - RD/C- Vehicle	-10.20	-12.174	-8.226	<0.001	<0.001
15	RD/C- 10 mg/kg - SD/C+ 10 mg/kg	1.20	-0.774	3.174	0.229	0.828
16	RD/C- 20 mg/kg - RD/C- Vehicle	-13.65	-15.744	-11.556	<0.001	<0.001
17	RD/C- 20 mg/kg - SD/C+ 10 mg/kg	-2.25	-4.344	-0.156	0.036	0.277
18	RD/C- Vehicle - SD/C+ 10 mg/kg	11.40	9.426	13.374	<0.001	<0.001

Model 23c: PWF Cold

Post Hoc analysis of Group Time interaction effect at 4 hours

Results to contrasts of group at 4 hours. Adjusted p-values are calculated using Tukey's Method.

	Contrast	Estimate	Lower CI	Upper CI	Unadjusted P-value	Ajusted P-value
7	RD/C- 10 mg/kg - RD/C- 20 mg/kg	1.6	-0.494	3.694	0.002	0.648
8	RD/C- 10 mg/kg - RD/C- Vehicle	-0.4	-2.374	1.574	<0.001	0.999
9	RD/C- 10 mg/kg - SD/C+ 10 mg/kg	3.4	1.426	5.374	0.229	0.013
10	RD/C- 20 mg/kg - RD/C- Vehicle	-2.0	-4.094	0.094	0.002	0.406
11	RD/C- 20 mg/kg - SD/C+ 10 mg/kg	1.8	-0.294	3.894	<0.001	0.525
12	RD/C- Vehicle - SD/C+ 10 mg/kg	3.8	1.826	5.774	0.229	0.004

Model 24a: Grip Force

	Df	Sum Sq	Mean Sq	F value	Pval
group	3	1387.947	462.649	15.006	<0.001
EN_time	4	3198.741	799.685	59.997	<0.001
group:EN_time	12	2163.778	180.315	59.997	<0.001

	Df	Sum Sq	Mean Sq	F value	Pval

Model 24b: Grip Force

Post Hoc analysis of Group Time interaction effect at 0.5 hours

Results to contrasts of of group at 0.5 hours. Adjusted p-values are calculated using Tukey's Method.

	Contrast	Estimate	Lower CI	Upper CI	Unadjusted P-value	Ajusted P-value
13	RD/C- 10 mg/kg - RD/C- 20 mg/kg	-8.533	-17.949	0.882	0.075	0.464
14	RD/C- 10 mg/kg - RD/C- Vehicle	16.600	7.723	25.477	<0.001	0.005
15	RD/C- 10 mg/kg - SD/C+ 10 mg/kg	-17.800	-26.677	-8.923	<0.001	0.002
16	RD/C- 20 mg/kg - RD/C- Vehicle	25.133	15.718	34.549	<0.001	<0.001
17	RD/C- 20 mg/kg - SD/C+ 10 mg/kg	-9.267	-18.682	0.149	0.054	0.371
18	RD/C- Vehicle - SD/C+ 10 mg/kg	-34.400	-43.277	-25.523	<0.001	<0.001

Model 24c: Grip Force

Post Hoc analysis of Group Time interaction effect at 4 hours

Results to contrasts of group at 4 hours. Adjusted p-values are calculated using Tukey's Method.

	Contrast	Estimate	Lower CI	Upper CI	Unadjusted P-value	Ajusted P-value
7	RD/C- 10 mg/kg - RD/C- 20 mg/kg	-7.117	-16.532	2.299	0.075	0.656
8	RD/C- 10 mg/kg - RD/C- Vehicle	-3.467	-12.344	5.410	<0.001	0.969
9	RD/C- 10 mg/kg - SD/C+ 10 mg/kg	-12.067	-20.944	-3.190	<0.001	0.087
10	RD/C- 20 mg/kg - RD/C- Vehicle	3.650	-5.766	13.066	0.075	0.970
11	RD/C- 20 mg/kg - SD/C+ 10 mg/kg	-4.950	-14.366	4.466	<0.001	0.897
12	RD/C- Vehicle - SD/C+ 10 mg/kg	-8.600	-17.477	0.277	<0.001	0.389
